# Behavioral Activation and Mindfulness Interventions in Reducing Loneliness and Improving Well-Being in Older Adults: The HEAL-HOA Randomized Clinical Trial

**DOI:** 10.1001/jamanetworkopen.2025.57170

**Published:** 2026-02-04

**Authors:** Vivien Foong Yee Tang, Da Jiang, Jojo Yan Yan Kwok, Dannii Yuen-lan Yeung, Namkee G. Choi, Lisa M. Warner, Rainbow Tin Hung Ho, Kee-Lee Chou

**Affiliations:** 1 Department of Social Sciences and Policy Studies, The Education University of Hong Kong, Tai Po, Hong Kong SAR, China; 2 Department of Special Education and Counselling, The Education University of Hong Kong, Tai Po, Hong Kong SAR, China; 3 School of Nursing, The University of Hong Kong, Pokfulam, Hong Kong SAR, China; 4 Centre on Behavioral Health, The University of Hong Kong, Pokfulam, Hong Kong SAR, China; 5 Department of Social and Behavioural Sciences, City University of Hong Kong, Hong Kong SAR, China; 6 School of Social Work, University of Texas at Austin, Austin; 7 Department of Psychology, MSB Medical School Berlin, Berlin, Germany; 8 Department of Social Work & Social Administration, Centre on Behavioral Health, The University of Hong Kong, Hong Kong SAR, China

## Abstract

**Importance:**

Late-life loneliness is a serious public health concern, yet the long-term efficacy and mechanisms of interventions to alleviate it remain underexplored.

**Objective:**

To evaluate the 12-month effectiveness of lay counselor-delivered, telephone-based behavioral activation and mindfulness interventions in reducing loneliness and enhancing well-being among at-risk older adults and to examine whether social isolation mediates these effects.

**Design, Setting, and Participants:**

The Helping Alleviate Loneliness in Hong Kong Older Adults (HEAL-HOA) was a 3-arm, assessor-blinded randomized clinical trial conducted from April 1, 2021, to April 28, 2024. Eligible participants 65 years or older who were living alone, digitally excluded, lonely, and experiencing financial hardship were recruited from public housing estates, community centers, academies for older adults, and word of mouth. Participants were randomized 1:1:1 to receive telephone-delivered behavioral activation (Tele-BA), telephone-delivered mindfulness (Tele-MF), or telephone-delivered befriending (Tele-BF [attention control]).

**Interventions:**

Participants received eight 30-minute sessions delivered by trained lay counselors via telephone for 4 weeks.

**Main Outcomes and Measures:**

The primary outcome was loneliness, measured using the UCLA Loneliness Scale (UCLA-LS) and the De Jong Gierveld Loneliness Scale (DJGL). Secondary outcomes included perceived stress, social support, sleep quality, life satisfaction, anxiety, depressive symptoms, and psychological well-being. Social isolation was examined as a mediator. Outcomes were assessed at baseline and at 1, 3, 6, and 12 months of follow-up.

**Results:**

Of 4152 individuals screened, 1151 participants (mean [SD] age, 76.6 [7.8] years; 843 [73.2%] female; 965 [83.8%] with ≥1 chronic illness) were randomized (Tele-BA, 335 [29.1%]; Tele-MF, 460 [40.0%]; Tele-BF, 356 [30.9%]). Intention-to-treat analyses using linear mixed-effects models showed significant between-group changes in loneliness at 12 months for Tele-BA (UCLA-LS: mean difference [MD], −0.73; 95% CI, −1.29 to −0.16; *P* = .01; DJGL: MD, −0.13; 95% CI, −0.23 to −0.03; *P* = .01) and Tele-MF (UCLA-LS: MD, −0.72; 95% CI, −1.24 to −0.20; *P* = .003) compared with Tele-BF. Secondary outcomes, including sleep quality, psychological well-being, and life satisfaction, also improved significantly. Social isolation at 6 months partially mediated the effect on loneliness (UCLA-LS) for both interventions at 12 months (accounting for 0.13 [13.5%] and 0.18 [18.0%] of the total effects for Tele-BA and Tele-MF, respectively).

**Conclusions and Relevance:**

In this randomized clinical trial, brief telephone-delivered behavioral activation and mindfulness interventions by lay counselors sustainably reduced loneliness and enhanced well-being during 12 months in at-risk older adults, with social isolation as a partial mediator. These scalable interventions offered effective solutions for combating late-life loneliness. Future research should investigate the applicability of these interventions outside the Hong Kong context as well as their cost-effectiveness.

**Trial Registration:**

Identifier: ChiCTR2300072909

## Key Points

**Question:**

Can 4-week, lay counselor, telephone-delivered interventions reduce loneliness in older adults for 12 months, and is this effect mediated by social isolation?

**Findings:**

In this dual randomized clinical trial of 1151 older adults who were living in poverty, alone, digitally excluded, and experiencing loneliness, telephone-delivered behavioral activation and mindfulness significantly reduced loneliness at 12 months compared with a befriending control. Social isolation at 6 months partially mediated the effects of both interventions on loneliness at 12 months in Hong Kong.

**Meaning:**

These results suggest that telephone-delivered behavioral activation and mindfulness interventions, provided by trained lay counselors, are scalable and effective for reducing loneliness and improving well-being in older adults, with sustained benefits throughout 12 months potentially enhanced by addressing social isolation.

## Teaser

This randomized clinical trial evaluates the effects of a 12-month telephone-delivered intervention by lay counselors using behavioral activation and mindfulness compared with telephone-delivered befriending on reducing loneliness among at-risk older adults.

## Introduction

Loneliness, a subjective gap between one’s desired and actual social relationships,^1^ is a pervasive global public health concern, affecting approximately 30% of older adults across the US, Europe, and Asia.^2^ In Hong Kong, approximately 46% of older adults reported feeling lonely in 2017.^3^ Loneliness is associated with poor cardiovascular, mental, and cognitive health and increased risk of premature mortality.^4,5,6,7^ The COVID-19 pandemic exacerbated these risks by limiting face-to-face interactions,^8,9,10^ highlighting the urgent need for scalable interventions to improve older adults’ well-being under highly restrictive conditions.

Previous research has explored diverse interventions aimed at reducing loneliness in older adults, including psychotherapies, physical activities, animal-assisted, and befriending services delivered in person, online, or by telephone.^11,12,13,14,15,16^ These interventions have been shown to reduce loneliness, with psychosocial approaches demonstrating comparatively greater effectiveness and both individual- and group-based formats appearing particularly beneficial.^11^ However, most studies have neither examined outcomes beyond 6 months nor elucidated interventions’ mechanisms, which are substantial gaps given the long-term and recurrent nature of loneliness.^17^ Establishing evidence of long-term effectiveness is essential for determining whether initial improvements are sustained, identifying the need for ongoing support, revealing the effects of delayed intervention, and informing policymakers about cost-effective and scalable solutions. Understanding underlying mechanisms helps identify active treatment components, explain response variability, and develop optimized, personalized interventions with greater precision and durability.

The Helping Alleviate Loneliness in Hong Kong Older Adults (HEAL-HOA) study, a unique 3-arm, parallel, dual randomized clinical trial conducted during the COVID-19 pandemic, addressed these gaps by repeatedly assessing loneliness and psychosocial factors in peer-delivered telephone interventions. The HEAL-HOA study demonstrated a significant reduction in loneliness at 1, 3, and 6 months,^18,19^ but long-term effects need to be examined. Social isolation, defined as the objective state of having few or infrequent social contacts, limited social roles, and reduced community engagement,^4,7^ is distinct from loneliness despite the overlap of these 2 concepts. Both are associated with substantial health risks.^20,21^ Recent studies^22,23^ suggested that social isolation may function as a mediator in loneliness intervention. Behavioral activation, which promotes engaging in rewarding and meaningful activities, has shown promise in reducing loneliness by decreasing social isolation.^24^ Mindfulness interventions, which foster present-moment awareness and acceptance, have been associated with increased daily social interactions and expanded social networks, thereby helping to alleviate loneliness.^25,26^ These approaches were selected because they target behavioral and emotional processes that can become self-reinforcing over time and have demonstrated benefits for mental health.^27,28^

Building on our earlier work, we evaluated the effects of a 12-month telephone-delivered behavioral activation (Tele-BA) and telephone-delivered mindfulness (Tele-MF) intervention provided by lay counselors compared with a telephone-delivered befriending (Tele-BF) intervention on reducing loneliness. Given the persistence of social isolation from COVID-19 restrictions, we also examined social isolation at 6-month follow-up as a mediator. This longitudinal approach aims to strengthen the causal inference drawn from previous analyses^18,19^ of the HEAL-HOA trial by examining intervention mechanisms during a 12-month period.

## Methods

### Study Design

**Figure 1.**
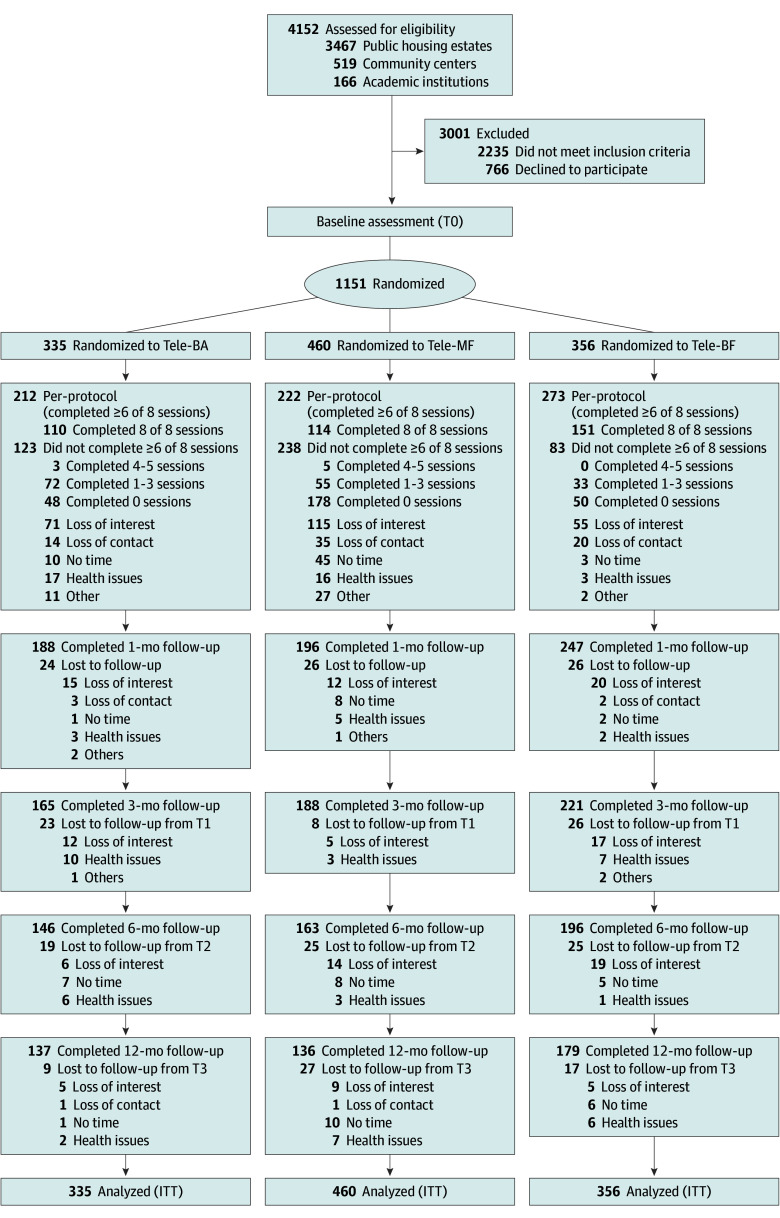
CONSORT Flow Diagram ITT indicates intention to treat; Tele-BA, telephone-delivered behavioral activation; Tele-MF, telephone-delivered mindfulness; Tele-BF, telephone-delivered befriending.

The HEAL-HOA trial was a 3-arm, parallel, assessor-blinded, dual randomized clinical trial conducted in Hong Kong from April 1, 2021, to April 28, 2024, to investigate the long-term effects of telephone-delivered psychosocial interventions delivered by lay counselors who were older Chinese retiree volunteers for lonely older adults who lived alone, were digitally excluded, and experienced financial difficulties.^29^ The trial protocol^30^ (Supplement 1) and short-term outcomes^18,19^ have been published elsewhere. The trial was approved by the Human Research Ethics Board of The Education University of Hong Kong, prospectively registered with the Clinical Trials Registry of The University of Hong Kong Clinical Trials Centre, and retrospectively registered with the Chinese Clinical Trial Registry, with identical content. The trial adhered to the Consolidated Standards of Reporting Trials (CONSORT) reporting guideline (Figure 1).

### Participants

Participants were recruited from public housing estates, community centers, academies for seniors, and word of mouth in Hong Kong between April 1, 2021, and April 30, 2023. Inclusion criteria were age of 65 years or older, experiencing loneliness (3-item UCLA Loneliness Scale [UCLA-LS] score ≥6), living alone, proficiency in Cantonese for telephone communication, lacking home internet access, and monthly income under US $577. Exclusion criteria were cognitive impairments, psychiatric disorders, suicide ideation (ie, participants were asked if they experienced any suicide ideation in the past 3 months), or regular engagement in mindfulness or other mind-body practices (>2 times weekly). Written informed consent was obtained, and participants were compensated with HK $250 (approximately US $31).

### Randomization and Blinding

After the baseline assessment, an independent research assistant used an online number generator to randomly assign participants in a 1:1:1 allocation ratio to Tele-BA, Tele-MF, or Tele-BF. Participants and lay counselors were not blinded to the allocation but remained unaware of the content of other interventions and the study hypotheses. Research assistants who conducted assessments were blinded to participants’ intervention assignments.

### Interventions

A total of 375 lay counselors aged 50 to 70 years who experienced loneliness were recruited, and 185 were randomly assigned to the volunteering condition, with 148 (80.0%) successfully delivering the interventions.^29,30,31^ All lay counselors received 12 hours of standardized, in-person training during 6 weeks (2 hours per week), focused specifically on the assigned intervention (Tele-BA or Tele-MF). Training included didactic instruction, practical exercises, and role-play sessions, followed by mock deliveries to ensure competence and consistency. Trained lay counselors delivered eight 30-minute telephone sessions twice a week to intervention participants for 4 weeks. Each lay counselor was randomly assigned 1 to 10 participants during a 6-month period.

The Tele-BA intervention, adapted from the *Telehealth Behavioral Activation Treatment Manual for Homebound Older Adults with Depression*,^32^ is a structured, goal-oriented program designed to reduce inactivity and avoidance behaviors while enhancing quality of life. It encourages participants to engage in meaningful activities and foster positive emotions by identifying key life areas, setting specific goals, selecting aligned activities, addressing challenges, and maintaining these practices independently beyond the intervention phase. Session details are provided in eTable 1 in Supplement 2.

The Tele-MF intervention, adapted from a smartphone mindfulness program for stressed adults, aimed to reduce loneliness by fostering present-moment awareness and acceptance.^25^ Techniques included body scanning, focused attention, relaxation methods, and cultivation of equanimity. Participants were guided to recognize bodily sensations and maintain a positive, accepting attitude toward stress. Session details are provided in eTable 2 in Supplement 2.

The Tele-BF intervention, adapted from the Befriends Information Guide by the National Aging Research Institute^33^ served as the attention control. Trained lay counselors offered emotional and informational support without teaching any psychosocial skills. Calls were personalized and flexible, based on participants’ interests (eg, latest news, vacation, and scam prevention), and provided supportive but nontherapeutic interactions.

### Fidelity and Monitoring

To ensure intervention fidelity, lay counselors completed structured record sheets documenting the session date, number, duration, and any challenges encountered. Continuous support was provided through WhatsApp group messaging, telephone calls, group sharing, and supervision sessions facilitated by the project team and trainers. Between January 1 and April 28, 2022, a notable increase in dropouts (n = 130) occurred among participants assigned to Tele-MF, primarily due to Hong Kong’s stringent social distancing measures. These restrictions disrupted in-person training sessions for lay counselors delivering the Tele-MF intervention, which in turn caused delays in participants’ receipt of the intervention, contributing to the elevated dropout rate. The targeted sample size of 322 participants per intervention arm was maintained by adjusting the recruitment ratio for Tele-BA, Tele-MF, and Tele-BF to 1:2:1 starting in April 2022, which resulted in an imbalanced sample distribution of 335:460:356.

### Measurement

Assessments, using validated Chinese versions, were conducted at 5 time points: baseline (T0), 1 month (T1; immediate effects), 3 (T2) and 6 (T3) months (short-term effects), and 12 months (T4; long-term effects), consistent with expectation that the intervention would reduce loneliness over time. Assessments at T0, T3, and T4 were conducted in-person, whereas T1 and T2 assessments were administered via telephone due to COVID-19 restrictions.

Demographic characteristics collected at baseline included age, gender, marital status, education level, number of children, and number of chronic illnesses. Loneliness was assessed using the revised UCLA Loneliness Scale (UCLA-LS) (score range, 20-80, with 20 indicating low level of loneliness and 80 indicating high level of loneliness)^34^ and the De Jong Gierveld Loneliness Scale (DJGL; score range, 0-6, with 0 indicating no loneliness and 6 indicating severe loneliness).^35^ Secondary outcomes included perceived stress (Perceived Stress Scale; score range, 0-56, with 0 indicating no perceived stress and 56 indicating extremely high perceived stress),^36^ perceived social support (Multidimensional Scale of Perceived Social Support; score range, 12-84, with 12 indicating low perceived social support and 84 indicating high perceived social support),^37^ sleep quality (Sleep Condition Indicator; score range, 0-32, with 0 indicating poor sleep quality and 32 indicating good sleep quality), life satisfaction (Satisfaction With Life Scale; score range, 5-35, with 5 indicating low life satisfaction and 35 indicating high life satisfaction),^38^ psychological well-being (Psychological Well-Being Scale; score range, 16-96, with 16 indicating low psychological well-being and 96 indicating high psychological well-being),^39^ depressive symptoms (Patient Health Questionnaire; score range, 0-27, with 0 indicating no depressive symptoms and 27 indicating severe depressive symptoms),^40^ and anxiety (Hospital Anxiety and Depression Scale anxiety subscale; score range, 0-21, with 0 indicating no anxiety and 21 indicating severe anxiety).^41^ The putative mediator was social isolation (Lubben Social Network Scale; score range, 0-30, with 0 indicating severe social isolation and 30 indicating strong social integration).^42^

### Sample Size

A previous study reported that a video-conferenced behavioral activation intervention reduced loneliness with a moderate effect size of 0.5 compared with a telephone-delivered friendly visit intervention.^43^ To be more conservative, this study powered its sample to detect smaller differences among 3 groups: Tele-BA, Tele-MF, and Tele-BF, assuming a small effect size of 0.25.^44^ Accordingly, 966 participants (322 per intervention group) were required for a 2-sided test at α = .05 and β = .10, while allowing 10% attrition over 6 months.

### Statistical Analysis

An intention-to-treat (ITT) analysis was performed for all randomized participants who completed the baseline assessment (N = 1151). Using SPSS software, version 29.0 (SPSS Inc), linear mixed modeling with random intercepts was used to estimate the interaction for time (T0 to T4) and group assignment (ie, Tele-BA vs Tele-BF and Tele-MF vs Tele-BF), adjusting for baseline loneliness and covariates (ie, age, gender, and marital status).^45^ Three models were analyzed: (1) ITT with baseline and covariate adjustments, (2) sensitivity analyses adjusting for baseline values of the outcomes, and (3) per-protocol analyses with baseline and covariates.^46^ Missing data were not imputed because mixed-effects models estimate missing values via direct likelihood methods.^47^ No missing data were observed among participants who completed the follow-up assessments. Sensitivity analyses using a pattern-mixture model were conducted to assess the potential impact of a missing-not-at-random mechanism.^48^ Family-wise type I error rate across the 4 primary 12-month comparisons (2 primary outcomes × 2 treatment contrasts) was controlled using the Holm-Bonferroni method, whereas secondary analyses used 2-sided, unadjusted *P* values, with *P* < .05 indicating statistical significance. Mediation of social isolation at 6 months (T3) on loneliness at 12 months (T4) was examined in R software, version 4.5.2 (R Foundation for Statistical Computing) using the mediation package with 5000 bootstrap samples to estimate the direct and indirect effects, adjusting for baseline and covariates, with sensitivity analyses assessing unmeasured mediator-outcome confounding.

## Results

Figure 1 illustrates the flow of participants through the study. A total of 4152 older adults were screened for eligibility via household visits in 102 public housing estates and referrals from 42 community centers and 4 academic institutions between April 1, 2021, and April 30, 2023. Of the 1917 eligible participants, 1151 consented and were randomized into 1 of the 2 intervention plus 1 control groups: Tele-BA (n = 335 [29.1%]), Tele-MF (n = 460 [40.0%]), and Tele-BF (n = 356 [30.9%]). Participant mean (SD) age was 76.6 (7.8) years; 843 (73.2%) were female and 308 (26.8%) were male; 932 (81.0%) were divorced or widowed; 863 (75.0%) had at least 1 child; and 965 (83.8%) reported at least 1 chronic illness (Table 1).

**Table 1.** Characteristics of the Sample at Baseline

Characteristic	No. (%) of participants		
	Tele-BA	Tele-MF	Tele-BF
No. (%)	335 (29.1)	460 (40.0)	356 (30.9)
Age, mean (SD), y	75.34 (7.01)	77.38 (7.97)	76.66 (8.12)
Gender			
Male	88 (26.3)	106 (23.0)	114 (32.0)
Female	247 (73.7)	354 (77.0)	242 (68.0)
Marital status			
Married	17 (5.1)	43 (9.3)	51 (14.3)
Divorced	115 (34.3)	263 (57.2)	93 (26.1)
Single (never married)	50 (14.9)	33 (7.2)	25 (7.0)
Widow	153 (45.7)	121 (26.3)	187 (52.5)
Educational level			
No education	75 (22.4)	169 (36.7)	90 (25.3)
Primary or below	118 (35.2)	184 (40.0)	146 (41.0)
Secondary	118 (35.2)	98 (21.3)	110 (30.9)
Tertiary	24 (7.2)	9 (2.0)	10 (2.8)
Children			
Without children	100 (29.9)	107 (23.3)	81 (22.8)
With children	235 (70.1)	353 (76.7)	275 (77.2)
Chronic illnesses^a^			
Did not have chronic illnesses	53 (15.8)	88 (19.1)	45 (12.6)
Had chronic illnesses	282 (84.2)	372 (80.9)	311 (87.4)
UCLA-LS score, mean (SD)^b^	50.43 (7.27)	51.63 (6.63)	51.54 (7.91)
DJGL score, mean (SD)^c^	4.50 (1.45)	4.83 (1.39)	4.28 (1.40)
PSS score, mean (SD)^d^	16.52 (11.69)	20.26 (10.82)	11.76 (11.12)
MSPSS score, mean (SD)^e^	40.96 (15.10)	37.88 (13.17)	38.06 (16.40)
SCI score, mean (SD)^f^	23.00 (7.67)	22.84 (7.74)	21.21 (8.48)
SWLS score, mean (SD)^g^	18.94 (4.09)	18.14 (4.04)	19.23 (3.54)
PWB score, mean (SD)^h^	56.90 (8.27)	55.13 (6.43)	55.92 (8.24)
PHQ-9 score, mean (SD)^i^	2.01 (3.52)	2.54 (3.95)	2.60 (3.97)
HADS-A score, mean (SD)^j^	1.75 (3.40)	2.53 (3.76)	2.26 (3.55)
LSNS score, mean (SD)^k^	8.15 (5.81)	7.20 (5.56)	8.46 (6.28)

Abbreviations: DJGL, De Jong Gierveld Loneliness Scale; HADS-A, Hospital Anxiety and Depression Scale anxiety subscale; LSNS, Lubben Social Network Scale; MSPSS, Multidimensional Scale of Perceived Social Support; PHQ-9, 9-item Patient Health Questionnaire; PSS, Perceived Stress Scale; PWB, Psychological Well-Being Scale; SCI, sleep condition indicator; SWLS, Satisfaction with Life Scale; Tele-BA, telephone-delivered behavioral activation; Tele-BF, telephone-delivered befriending; Tele-MF, telephone-delivered mindfulness; UCLA-LS, UCLA Loneliness Scale.

^a^ Chronic nonspecific lung diseases, cardiac disease, peripheral disease, stroke, diabetes, arthritis, and cancer.

^b^ UCLA-LS scores range from 20 to 80, with 20 indicating low level of loneliness and 80 indicating high level of loneliness.

^c^ DJGL scores range from 0 to 6, with 0 indicating no loneliness and 6 indicating severe loneliness.

^d^ PSS scores range from 0 to 56, with 0 indicating no perceived stress and 56 indicating extremely high perceived stress.

^e^ MSPSS scores range from 12 to 84, with 12 indicating low perceived social support and 84 indicating high perceived social support.

^f^ SCI scores range from 0 to 32, with 0 indicating poor sleep quality and 32 indicating good sleep quality.

^g^ SWLS scores range from 5 to 35, with 5 indicating low life satisfaction and 35 indicating high life satisfaction.

^h^ PWB scores range from 16 to 96, with 16 indicating low psychological well-being and 96 indicating high psychological well-being.

^i^ PHQ-9 scores range from 0 to 27, with 0 indicating no depressive symptoms and 27 indicating severe depressive symptoms.

^j^ HADS-A scores range from 0 to 21, with 0 indicating no anxiety and 21 indicating severe anxiety.

^k^ LSNS scores range from 0 to 30, with 0 indicating severe social isolation and 30 indicating strong social integration.

At 12 months, 699 participants (60.7%) had dropped out: 198 (59.1%) in the Tele-BA group, 324 (70.4%) in the Tele-MF group, and 177 (49.7%) in the Tele-BF group (Figure 1). Completers (n = 452) and noncompleters (n = 699) were similar across most demographics and baseline characteristics, although completers had lower educational attainment (eTable 3 in Supplement 2). Among participants who completed the follow-up assessments, no data were missing. Pattern-mixture model analyses showed no significant interactions between missing data patterns and time.

### Intervention Adherence

Among 1151 randomized participants, 707 (61.4%) completed 6 or more sessions of the assigned intervention: 212 (63.3%) in the Tele-BA group, 222 (48.3%) in the Tele-MF group, and 273 (76.7%) in the Tele-BF group. Reasons for nonadherence included loss of interest, loss of contact, no time, health issues, and other personal circumstances (Figure 1). Nonadherence was higher in the Tele-MF group, aligning with periods of strict social distancing that disrupted lay counselor training and delayed intervention delivery.

### Primary Outcome

Table 2 presents the results of the ITT analyses. At 12 months, both loneliness measures showed statistically significant overall between-group differences after Holm-Bonferroni correction. For UCLA-LS, the group × time interaction showed a significant reduction in loneliness for Tele-BA (mean difference [MD], −0.73; 95% CI, −1.29 to −0.16; Cohen *d* = 0.11; Holm-adjusted *P* = .02) and Tele-MF (MD, −0.72; 95% CI, −1.24 to −0.20; Cohen *d* = 0.11; Holm-adjusted *P* = .01) when compared with Tele-BF. For DJGL, the group × time interaction revealed a significant reduction in loneliness for Tele-BA (MD, −0.13; 95% CI, −0.23 to −0.03; Cohen *d* = 0.09; Holm-adjusted *P* = .02), whereas Tele-MF did not (MD, −0.03; 95% CI, −0.13 to 0.06; Cohen *d* = 0.07; Holm-adjusted *P* > .99) compared with Tele-BF. To enhance clinical interpretation, we applied a prespecified minimal clinically important difference threshold of a 2.86-point or greater reduction on the UCLA-LS from baseline to 12 months.^49^ Among participants achieving this level of improvement, moderate and clinically meaningful effect sizes were observed (Tele-BA: *d* = 0.35; Tele-MF: *d* = 0.43) compared with Tele-BF.

**Table 2.** Mixed-Effects Analysis at 12 Months Using Intention to Treat^a^

Measure	Estimates across assessments, mean (95% CI)^b^						Estimates vs Tele-BF						Overall between-group difference *P* value
							Tele-BA			Tele-MF			
	Tele-BA (n = 335)	*P* value	Tele-MF (n = 460)	*P* value	Tele-BF (n = 356)	*P* value	MD (95% CI)	Cohen *d*	MD *P* value	MD (95% CI)	Cohen *d*	MD *P* value	
Primary outcomes													
UCLA-LS^c^	48.04 (47.66 to 48.42)	<.001	48.04 (47.71 to 48.38)	<.001	48.76 (48.41 to 49.12)	<.001	−0.73 (−1.29 to −0.16)	0.11	.01	−0.72 (−1.24 to −0.20)	0.11	.003	<.001
DJGL^d^	3.87 (3.81 to 3.94)	<.001	3.97 (3.91 to 4.03)	<.001	4.00 (3.94 to 4.07)	<.001	−0.13 (−0.23 to −0.03)	0.09	.01	−0.03 (−0.13 to 0.06)	0.07	>.99	<.001
Secondary outcomes													
SCI^e^	22.48 (22.11 to 22.84)	<.001	22.38 (22.06 to 22.70)	<.001	22.05 (21.71 to 22.40)	.51	0.42 (−0.12 to 0.97)	0.06	>.99	0.33 (−0.18 to 0.83)	0.08	.37	<.001
PWB^f^	60.22 (59.83 to 60.61)	<.001	59.73 (59.38 to 60.07)	<.001	59.76 (59.39 to 60.13)	<.001	0.46 (−0.13 to 1.04)	0.14	.18	−0.04 (−0.58 to 0.50)	0.14	>.99	<.001
MSPSS^g^	43.49 (42.77 to 44.20)	<.001	43.43 (42.79 to 44.06)	<.001	43.06 (42.38 to 43.74)	<.001	0.43 (−0.64 to 1.50)	0.11	>.99	0.37 (−0.62 to 1.36)	0.17	>.99	<.001
PHQ-9^h^	2.39 (2.22 to 2.56)	.87	2.49 (2.34 to 2.65)	.02	2.62 (2.45 to 2.78)	.01	−0.23 (−0.49 to 0.03)	0.34	.11	−0.12 (−0.36 to 0.12)	0.33	.67	<.001
HADS-A^i^	2.12 (1.99 to 2.26)	.42	2.33 (2.22 to 2.44)	.01	2.29 (2.16 to 2.42)	.002	−0.17 (−0.39 to 0.06)	0.33	.23	0.04 (−0.17 to 0.25)	0.30	>.99	<.001
PSS^j^	14.97 (14.52 to 15.42)	<.001	15.55 (15.15 to 15.95)	.92	14.98 (14.54 to 15.42)	<.001	−0.01 (−0.69 to 0.67)	0.02	>.99	0.57 (−0.08 to 1.23)	0.21	>.99	<.001
SWLS^k^	20.74 (20.54 to 20.94)	<.001	20.54 (20.37 to 20.72)	<.001	20.50 (20.32 to 20.69)	<.001	0.23 (−0.06 to 0.53)	0.13	.18	0.04 (−0.24 to 0.32)	0.15	>.99	<.001

Abbreviations: DJGL, De Jong Gierveld Loneliness Scale; HADS-A, Hospital Anxiety and Depression Scale anxiety subscale; MD, mean difference; MSPSS, Multidimensional Scale of Perceived Social Support; PHQ-9, 9-item Patient Health Questionnaire; PSS, Perceived Stress Scale; PWB, Psychological Well-Being Scale; SCI, sleep condition indicator; SWLS, Satisfaction With Life Scale; Tele-BA, telephone-delivered behavioral activation; Tele-BF, telephone-delivered befriending; Tele-MF, telephone-delivered mindfulness; UCLA-LS, UCLA Loneliness Scale.

^a^ Linear mixed models with maximum likelihood missing data treatment, controlling for age, gender, marital status, and baseline outcome. All *P* values reported are nonadjusted. *P* ≤ .01 is considered statistically significant for primary outcomes.

^b^ Paired *t* test between scores at baseline and other assessment points were conducted for each intervention group separately. Results at 12 months are shown here.

^c^ UCLA-LS scores range from 20 to 80, with 20 indicating low level of loneliness and 80 indicating high level of loneliness.

^d^ DJGL scores range from 0 to 6, with 0 indicating no loneliness and 6 indicating severe loneliness.

^j^ PSS scores range from 0 to 56, with 0 indicating no perceived stress and 56 indicating extremely high perceived stress.

^g^ MSPSS scores range from 12 to 84, with 12 indicating low perceived social support and 84 indicating high perceived social support.

^e^ SCI scores range from 0 to 32, with 0 indicating poor sleep quality and 32 indicating good sleep quality.

^k^ SWLS scores range from 5 to 35, with 5 indicating low life satisfaction and 35 indicating high life satisfaction.

^f^ PWB scores range from 16 to 96, with 16 indicating low psychological well-being and 96 indicating high psychological well-being.

^h^ PHQ-9 scores range from 0 to 27, with 0 indicating no depressive symptoms and 27 indicating severe depressive symptom.

^i^ HADS-A scores range from 0 to 21, with 0 indicating no anxiety and 21 indicating severe anxiety.

Results from the sensitivity analysis, which adjusted for the baseline value of the primary outcome, were consistent with the main findings (eTable 4 in Supplement 2). For the per-protocol analyses (eTable 5 in Supplement 2), the overall between-group differences remained significant; however, the group × time interaction for Tele-BA and Tele-MF relative to Tele-BF was nonsignificant at all time points.

### Mediating Effect of Social Isolation on Loneliness

**Figure 2.**
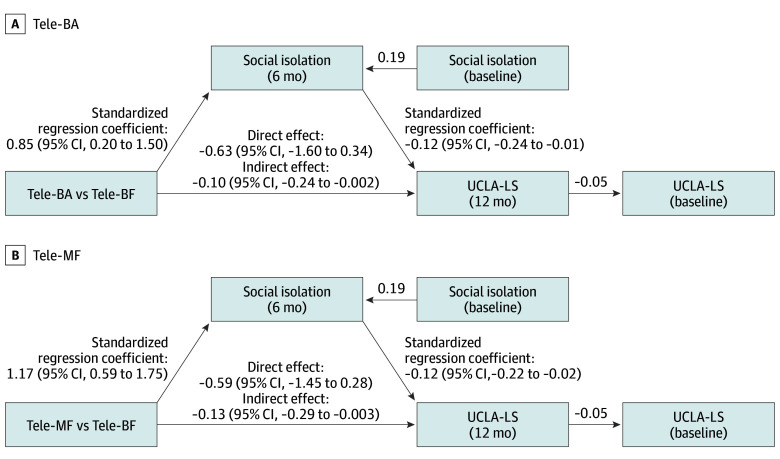
Mediating Effect of Social Isolation on Loneliness All analyses are controlled for baseline social isolation, baseline loneliness, age, gender, and marital status. Tele-BA indicates telephone-delivered behavioral activation; Tele-BF, telephone-delivered befriending; Tele-MF, telephone-delivered mindfulness; UCLA-LS, UCLA Loneliness Scale.

Mediation analyses were used to examine whether social isolation at 6 months (T3) mediated the effects of Tele-BA and Tele-MF interventions, compared with Tele-BF, on loneliness at 12 months (T4), adjusting for baseline loneliness, baseline social isolation, age, gender, and marital status. Social isolation at T3 partially mediated the intervention effects on loneliness. Participants in Tele-BA and Tele-MF groups reported significantly lower levels of social isolation at T3 than those in Tele-BF (Tele-BA: β = 0.85, 95% CI, 0.20-1.50; *P* = .001; Tele-MF: β = 1.17; 95% CI, 0.59-1.75; *P* < .001), and reductions in social isolation were associated with reduced loneliness measured by UCLA-LS at T4 (Tele-BA: β = −0.12; 95% CI, −0.24 to −0.01; *P* = .03; Tele-MF: β = −0.12; 95% CI, −0.22 to −0.02; *P* = .02). The indirect effect of social isolation was significant for Tele-BA (β = −0.10; 95% CI, −0.24 to −0.002; *P* = .047) and Tele-MF (β = -0.13; 95% CI, −0.29 to −0.003; *P* = .04), accounting for 0.13 (13.5%) and 0.18 (18.0%) of the total effects, respectively (Figure 2). Direct effects were negative but not statistically significant for Tele-BA (β = −0.63; 95% CI, −1.60 to 0.34; *P* = .21) and Tele-MF (β = −0.59; 95% CI, −1.45 to 0.28; *P* = .18). Sensitivity analyses using a ρ-based approach indicated that the indirect effect would be reduced at a residual correlation of −0.10 (*R*^2^ = 0.01), reflecting minimal unmeasured confounding. The outcome models explained a small proportion of variance (*R*^2^ = 0.02). However, no significant indirect effect was found for DJGL in either Tele-BA (β = −0.01; 95% CI, −0.03 to 0.01; *P* = .42) or Tele-MF (β = −0.01; 95% CI, −0.03 to 0.02; *P* = .48).

### Secondary Outcomes

Table 2 and eTable 4 and eTable 5 in Supplement 2 presents the ITT, sensitivity, and per-protocol analyses. At the 12-month follow-up, significant overall between-group differences showed better sleep quality, psychological well-being, perceived social support, and life satisfaction, as well as lower levels of depressive symptoms, anxiety, and perceived stress (overall between-group difference *P *< .001).

### Safety and Adverse Events

During the randomized clinical trial, 2 participants in the Tele-MF group died. These deaths were unrelated to the trial intervention because participants continued to have full access to their preferred health care services and lifestyle choices.

## Discussion

Conducted as part of the HEAL-HOA dual randomized clinical trial, this study, to our knowledge, is the largest to date and among the first to demonstrate measurable 12-month outcomes of 2 scalable, lay counselor, telephone-delivered interventions: behavioral activation and mindfulness compared with an attention control receiving a befriending intervention. Our findings support the feasibility and scalability of trained older lay counselors as deliverers of remote interventions, consistent with previous research^18,19,50,51^ demonstrating the effectiveness and practicality of approaches to enhancing well-being led by older lay counselors. This approach leverages peer relatability, which increases engagement in older adults, while simultaneously addressing systemic barriers, such as the shortage of specialized clinicians. Moreover, it provides a sustainable solution for broadening access to psychosocial support in aging populations, particularly in low-resource and hard-to-reach settings.

Although most previous studies^52^ have examined only short-term outcomes, our findings provide evidence of the long-term benefits of brief Tele-BA and Tele-MF interventions, without booster sessions, in reducing loneliness. At 12-month follow-up, Tele-BA produced significant improvements on both the UCLA-LS and DJGL, whereas Tele-MF demonstrated significant effects on the UCLA-LS only. Although these between-group differences reached statistical significance, the small effect sizes suggest that the practical impact of these changes in daily functioning may be modest. Nevertheless, the presence of substantial and meaningful benefits among a subset of participants highlights the heterogeneity in treatment response. These findings underscore the importance of developing adaptive, personalized, and sustained intervention approaches to better support nonresponders and maintain treatment gains over time.

Additionally, the overall between-group differences reported improved sleep quality and perceived social support, enhanced psychological well-being and life satisfaction, and reduced depressive symptoms, anxiety, and perceived stress. We also found that the significant indirect and nonsignificant direct effects indicate that Tele-BA and Tele-MF reduce loneliness primarily by alleviating social isolation, underscoring social isolation as a key mechanism of change.

The differing effects of Tele-BA and Tele-MF on secondary outcomes likely reflect their distinct mechanisms, despite being effective in reducing loneliness. Tele-BA, rooted in research on behavioral activation, combats loneliness by adopting a more positive attitude toward social connections and facilitating meaningful behavioral changes. It emphasizes enhancing self-efficacy, which motivates individuals to engage in goal-directed activities that are fulfilling and purposeful, which strengthens social connections and a greater sense of purpose.^14,27^ This aligns with previous studies that emphasized that behavioral activation interventions effectively boost self-efficacy, encouraging social participation and reducing loneliness while improving mental and emotional well-being.^53,32^ Tele-MF, based on evidence of mindfulness and acceptance techniques, helps participants regulate negative emotions and maladaptive cognitive patterns associated with loneliness. By cultivating a peaceful and nonjudgmental attitude toward oneself, Tele-MF enhances emotional regulation and resilience toward social isolation.^54,55,56^ Mindfulness interventions have been shown to improve social cognition by increasing awareness toward positive social cues and prompting the mobilization of social support.^25,57^ Furthermore, social isolation mediated the effect of both interventions on loneliness, suggesting that despite different pathways, Tele-BA and Tele-MF share the core mechanism of enhancing participants’ ability to foster social connection. However, some divergent effects on loneliness may also reflect differences in measures and time points across the interventions, which were not directly examined in this study.

It is also important to consider the contextual influence of the easing of the strictest COVID-19 restrictions, which coincided with the period between 3 months (T2) and 6 months (T3) of our follow-up assessments. The relaxation of social distancing measures may have enabled participants to reconnect with their communities and social networks, further strengthening the skills and strategies they developed during the Tele-BA and Tele-MF intervention. By the 12-month follow-up (T4), public health measures had largely stabilized, with most restrictions lifted, allowing for increased social interaction and access to community resources. The broader return to normalcy likely supported sustained improvements in participants’ well-being and may have contributed to the positive direct intervention as well as mediation effects via lower levels of social isolation observed in our study. Future studies may want to investigate more targeted approaches to mitigate social isolation by enhancing group-based social identity and connections.^58,59,60^ Such strategies could be particularly beneficial during a pandemic or for at-risk populations experiencing physical or social immobilization. Although the long-term impact and mediation were not detected by the DJGL scale, this may be explained by the DJGL’s potential limitations in capturing gradual changes over time.^61^ Previous research^62,63^ highlighted that the DJGL failed to identify significant reductions in loneliness at 6 months after a lifestyle intervention as well as 12 months after a friendship program. Another possible reason could lie in the conceptual differences between the UCLA-LS and DJGL. Unlike the UCLA-LS, which assesses individuals’ current feelings of individuals, the DJGL captures more general perceptions of loneliness, which may be less sensitive to subtle changes over time.^64,65^

### Strengths and Limitations

This randomized clinical trial has several strengths. It included a large sample of vulnerable older adults and assessed long-term outcomes at 12 months conducted during the COVID-19 pandemic, which strengthened the robustness of the findings and their applicability to similar at-risk population. Beyond the COVID-19 pandemic, both Tele-BA and Tele-MF were replicated in a separate study with a similar population and demonstrated consistent effectiveness in reducing loneliness.

However, this study also has limitations. These findings were mainly applicable to lonely, Cantonese-speaking, older adults with limited resources and may not be generalizable to the broader populations with greater socioeconomic, cultural, or linguistic diversity. Although participants were randomly assigned to trained lay counselors, minor clustering effects due to individual differences in counselor skill or fidelity cannot be entirely excluded. However, sensitivity analyses, including counselor as a random effect, indicated an intraclass correlation below 0.01, suggesting negligible influence on the findings. Future research could adapt and evaluate these interventions in more diverse populations with varying languages and explore strategies to minimize lay counselor variability.

## Conclusions

In this randomized clinical trial conducted within the HEAL-HOA trial, brief, telephone-based psychosocial interventions delivered by trained lay counselors effectively reduced loneliness and enhanced social support, sleep quality, and psychological well-being among socially at-risk older adults in Hong Kong, both in the short and long term up to 12 months of follow-up. Reductions in loneliness were associated with participants’ active engagement in maintaining social connections. The involvement of older adult peers as lay counselors underscored the potential of these interventions to offer accessible, scalable solutions for promoting well-being in resource-limited contexts. Future research should evaluate the cost-effectiveness and implementation feasibility of these approaches across diverse populations and refine adaptive intervention sequences to enhance and sustain long-term outcomes.

## Notes

Supplement 1.
Trial Protocol

Supplement 2. **eTable 1. **Session Content of Tele-BA **eTable 2.** Session Content of Tele-MF **eTable 3. **Characteristics of Completers and Non-Completers at Baseline **eTable 4**. Mixed-Effects Analysis at 12 months (Sensitivity) **eTable 5**. Mixed-Effects Analysis at 12 months (Per-protocol)

Supplement 3.
Data Sharing Statement
