# Supplementary material for: Behavioral Activation and Mindfulness Interventions in Reducing Loneliness and Improving Well-Being in Older Adults: The HEAL-HOA Randomized Clinical Trial
Source: JAMA Netw Open. 2026 Feb 4;9(2):e2557170. doi: 10.1001/jamanetworkopen.2025.57170 (PMC12873770; doi:10.1001/jamanetworkopen.2025.57170)
Supplement: Supplement 1. — Trial Protocol [file jamanetwopen-e2557170-s001.pdf]

Supplement 1. Study Protocol

Title: Study Protocol of an RCT Comparing Telephone-delivered Behavioral Activation and Mindfulness Interventions against Befriending Intervention to Reduce Loneliness among Older Adults

Running head: Behavioral Activation and Mindfulness Intervention to Reduce Loneliness

Abstract

Background

Loneliness is one of the most prevalent global mental health problems among older adults and has become particularly concerning during the COVID-19 pandemic with constraints on social engagement. To reduce loneliness in older adults, we will examine the effectiveness of two telephone-delivered loneliness interventions (i.e., behavioral activation based on the behavioral theory of depression and mindfulness based on the monitor and acceptance theory) against a telephone-delivered befriending intervention as an active control. To meet the demand for loneliness-reducing interventions and enhance sustainability and scalability, retirees are recruited and trained as volunteers to implement these interventions.

Methods

In a three-arm randomized controlled trial, older adults in Hong Kong who live alone and under the poverty line, do not have Internet at home, and feel lonely will be randomly assigned to receive one of the three interventions (enrollment goal, $N = 966$). The primary outcomes will be loneliness, social network indicators and perceived social support. Secondary outcomes will be perceived stress, sleep quality, anxiety and depressive symptoms, physical and cognitive health. Underlying mechanisms will be examined with mindfulness, activity happiness/meaningfulness, diurnal cortisol patterns, levels of the salivary C Reactive Protein, and genetic markers (candidate gene, GWAS, biological pathway related SNPs, and polygenic risk scores).

Discussion

The findings are expected to provide robust evidence for the effectiveness of behavioral activation and mindfulness interventions to reduce loneliness and improve well-being among older adults in Hong Kong. Delivery via telephone facilitates scalability and sustainability and will allow large-scale implementations.

The study was prospectively registered in the Clinical Trials Registry of the University of Hong Kong Clinical Trials Centre (registration number: HKUCTR-2929) and retrospectively registered at the Chinese Clinical Trial Registry in the WHO Registry Network (number ChiCTR2300072909). The contents of the two registrations are identical.

Supplement 1. Study Protocol

Background

Loneliness is one of the most prevalent psychological problems among older adults globally. Studies have shown that loneliness affects more than 20% of older adults in China, Europe, and the United States (1–3). Loneliness in older adults is linked to various age-related risk factors, such as functional impairments in daily activities or social isolation (4), as well as to poor mental health outcomes, including depressive symptoms (Cacioppo et al., 2006) or suicidal ideation (6). The COVID-19 pandemic has aggravated risks for loneliness (7,8), since physical distancing measures aimed at reducing transmission of the SARS-CoV-2 virus restricted older adults' participation in social activities and reduced their contact with family members and friends. There is an urgent need to develop effective interventions to help reduce loneliness among community-dwelling older adults, especially those who live alone and have limited access to the Internet (i.e., social media connections) in future crisis situations.

Research has shown two effective approaches to reducing loneliness (9–11) that are grounded in theories of bipolarities of emotion and emotion regulation (12,13). The first approach focuses on improving positive affect (14,15) by increasing access to social interactions and social support and enhancing social skills via, for example, activity-based group interventions (16). An intervention strategy within this approach is Behavioral activation (BA). BA is designed to help individuals engage in meaningful and pleasant activities and to avoid aversive stimuli and has been found effective in reducing loneliness and depression in older adults (17,18). The second approach focuses on supporting individuals to better regulate negative emotions (e.g., stress and anxiety) triggered by loneliness (19,20) and making them aware of possible maladaptive cognitions in social situations (21). For example, mindfulness meditation interventions (MF) have been shown to be effective in reducing loneliness by guiding participants to focus on present-moment experiences, accept negative experiences, and remain equanimous (20). However, scientific evidence regarding the most effective approaches and their scalability and sustainability need to be firmly established (22). To address this research gap, we aimed at comparing these two approaches by examining the effectiveness of telephone-delivered BA and MF against simple befriending as an active control. Here we provide the protocol of the interventions and study design to test them.

Behavioral Activation

Building on the behavioral explanations of learning theory, BA posits that symptoms of depression will decrease when one engages in positively reinforced daily activities (i.e., healthy behaviors) (23–26). BA is a brief, uncomplicated, and structured treatment of depression (25,27), and its effectiveness has been found to be comparable to the effect of cognitive behavioral therapy, but at a lower cost (28).

Because of its observed benefits in alleviating depressive symptoms, BA has also been applied to nonclinical samples. For example, using a non-age-specific sample, studies have demonstrated the effectiveness of BA in reducing participants' level of loneliness, both when BA is administered separately (29) or as part of a

larger intervention package (30). BA has also been used to reduce loneliness in older adults (see Orgeta et al., 2017 for a meta-analysis). Choi and colleagues, for example, developed a videoconferencing BA program for low-income, housebound, lonely older Americans (31). The intervention consisted of five one-hour weekly sessions delivered remotely, with the aims of promoting engagement in activities aligned with older adults' personal values, reviewing their daily activity patterns, and planning and executing activity goals. Compared with the participants in the active control (friendly visit) group, the participants in the BA intervention engaged in more social interactions, felt more satisfied with social support, and reported lower levels of loneliness, depression, and disability (31). Similar intervention programs with BA features have also been shown to be effective in reducing older adults' loneliness and depressive symptoms in England and Wales (32) as well as in Sweden (33). To sum up, BA is designed to increase engagement in meaningful and pleasant activities through (a) behavior monitoring and activity planning; (b) the use of behavioral strategies such as relaxation, stress management, and mastery; and (c) the enhancement of social skills such as seeking social support from others (26,31).

Mindfulness Intervention

MF interventions aim to cultivate participants' present-moment focus with acceptance and nonjudgmental awareness (20). This focus is particularly effective in regulating the negative emotions triggered by loneliness (34). MF interventions have been shown to be effective in improving interpersonal relationship skills and relationship quality (35), fostering emotional regulation (36) and compassion toward others (37), improving social relationship functioning in couples (38), and in reducing anxiety, depressive symptoms (39) and loneliness (40).

In a review of 15 MF interventions, Geiger and colleagues demonstrated the feasibility and acceptability of using MF interventions in older adults (41). This review highlights that all included studies found MF interventions to be effective in promoting physical and emotional well-being among older adults (41). However, few interventions have focused on loneliness and interpersonal outcomes in older adults. In a small randomized controlled trial (RCT) of 40 healthy older adults (19), the face-to-face Mindfulness-Based Stress Reduction (MBSR) program, consisting of eight weekly 120-minute group sessions, a day-long retreat, and a daily 30-minute home practice, reduced loneliness. Creswell et al. also showed a reduction in C-reactive protein (CRP) to a marginally significant level. Kwok et al. conducted a weekly 90-minute MF yoga intervention over 8 weeks in Hong Kong, and showed that this intervention was effective in reducing anxiety and depressive symptoms in adults with Parkinson's disease, most of whom were older adults (39). All of these interventions were delivered face to face. In another small-scaled feasibility study conducted in Hong Kong. Kwok et al. examined an eight biweekly 90-min home-based MF intervention delivered via video-conferencing software (Zoom), and revealed that the telehealth approach was feasible, safe, and well-accepted among older adults, particularly in relieving

the burden brought by COVID-19 pandemic. The study findings highlighted the potentials of delivering psychosocial interventions through telehealth approach (36).

In their Monitor and Acceptance Theory (MAT), Lindsay and Creswell (2017) posit that MF increases cognitive functioning by enhancing individuals' awareness of their experiences and reduces negative affective reactivity by increasing acceptance (42). Based on MAT, Lindsay et al. developed a 2-week smartphone-based MF RCT with 14 daily sessions and tested it in an adult sample in the US. This intervention effectively reduced loneliness by 22% and increased social interaction by 2 times per day among the participants (20). However, the participants in this RCT were not older adults. There was also no follow-up after the completion of the intervention, which makes it difficult to draw conclusions about its long-term effectiveness. Therefore, we aim to evaluate the effectiveness of a telephone-delivered MF (Tele-MF) intervention in reducing loneliness among older adults in Hong Kong by adopting the intervention materials from Lindsay et al. (20). We aim to examine the short- and long-term effectiveness of the intervention with follow-up assessments at 4 weeks, 3 months, and 6 months post-intervention.

Delivering an Intervention by Telephone and by Trained Lay Volunteers

There are significant challenges to in-person delivery of interventions to reduce loneliness in older adults. Many older adults face transportation problems. Deploying interventionists for in-home sessions with older adults is very costly, and therefore not sustainable and scalable. In light of these challenges of in-person delivery, recent intervention programs have used technology as a way to reduce loneliness and social isolation in older adults (see (43) for a scoping review). In addition to the aforementioned examples by Choi et al (2020) and Lindsay et al. (2019), Tsai et al. (2020) also developed a smartphone-based videoconferencing program allowing nursing home residents to contact their families through video calls in Taiwan (44). The results of this 6-month intervention study revealed that the level of loneliness of residents in the intervention group significantly reduced compared to that of residents in the control group. Older veterans who received a telephone-delivered social activity intervention, as well as older adults who received telephone-delivered BA for depression during the COVID-19 pandemic rated telephone delivery as highly acceptable (33,45). Given the high contagiousness of the SARS-CoV-2 virus, use of telephone or videoconferencing has been found to be an especially useful and inexpensive approach to reducing the levels of loneliness and social isolation of older adults during the pandemic when social/physical distancing measures were in place (43).

Along with telephone delivery, nonprofessional delivery of mental health interventions is an important area of research given the global shortages of professional mental health workers. Unlike other psychotherapeutic approaches that rely heavily on mental health clinicians, studies have demonstrated that BA can be delivered by trained volunteers or lay counselors with a bachelor's degree (17,46–48). Therefore, we proposed to compare the effectiveness of telephone-delivered BA and MF interventions (hence Tele-BA and Tele-MF) by trained volunteers (see study protocol for volunteers part for the trial Warner et al., under

review(49) to that of telephone-delivered befriending (Tele-BF, i.e., providing emotional support) (50,51) as an active control in reducing loneliness in older adults.

Study Objectives and Hypothesis

The COVID-19 pandemic has undoubtedly imposed a wide range of restrictions on older adults' participation in social activities, increasing their level of loneliness. Low-income older adults who live alone and have limited access to the Internet are at higher risk of loneliness (52,53). Our primary aim is to evaluate whether a Tele-Behavioral Activation (Tele-BA) and a Tele-Mindfulness (Tele-MF) intervention are more effective than the active control consisting of Tele-Befriending (Tele-BF) in reducing loneliness (i.e., one of the primary outcomes, see below for details on all measures) among community-dwelling older Chinese adults in Hong Kong who live alone and in poverty and have limited access to the Internet. Tele-BA focuses on increasing and reinforcing a target individual's engagement in meaningful life activities (31), Tele-MF focuses on increasing awareness of the present moment with acceptance (20), whereas Tele-BF refers to a volunteer's provision of emotional support for the target individual who reports loneliness (50). We hypothesize that Tele-BA and Tele-MF interventions are effective in reducing perceived loneliness, measured at the 4-week, 3-month, 6-month, and 12-month follow-up assessments, compared to Tele-BF group.

Method/design

Research Integrity

Ethical approval for this proposed project was obtained from the Human Research Ethics Committee of the Education University of Hong Kong (reference number: 2019-2020-0442). The study was prospectively registered in the Clinical Trials Registry of the University of Hong Kong Clinical Trials Centre (HKU-CTC) (registration number: HKUCTR-2929) and retrospectively registered at the Chinese Clinical Trial Registry in the WHO Registry Network, number ChiCTR2300072909). The contents of the two registrations were identical.

Participant Recruitment and Eligibility

This study protocol targets community-dwelling Chinese older adults. The inclusion criteria are as follows: (a) age 65 and older; (b) living alone; (c) living in poverty (i.e., monthly income < HKD 4,500 or USD 577); (d) able to communicate in Cantonese by telephone; (e) mild or no cognitive impairment; (f) no psychiatric disorder; (g) no Internet at home; (h) not engaged in regular mindfulness or a related mind–body practice; and (i) feeling lonely (score of 6 or higher on the three-item UCLA Loneliness Scale, Lin et al., 2020). Participants have been recruited through community centers for older adults, older-adult academies, and from public housing estates. The study's purpose, time commitment for participation, voluntariness, right to quit without negative consequences, and inclusion criteria have been clearly presented before a participant gives verbal consent to participate. Older adults who meet the above criteria have then been asked to sign a written informed consent form. The current stage of the study is that interventions and follow-up assessments are under way.

Supplement 1. Study Protocol

Sample Size

A required sample size for the RCT on loneliness was estimated for one of the primary outcomes, that is, loneliness assessed by UCLA Loneliness Scale (54). A previous intervention study on reducing loneliness in older adults yielded an effect size of 0.5 (31). To be more conservative, we estimate the sample size based on a smaller effect size, i.e., 0.25. To detect an effect size of 0.25 at an alpha error rate of 0.05 and a power of 90%, the estimated sample size is 289 participants in each group. With an anticipated drop-out rate of 10% over a period of 6 months, the necessary sample size is 322 participants per group at baseline (*N*=966 in total).

Study Design

Participants are interviewed by trained assessors using structured questionnaires at five time points: at baseline (pre-intervention) and at 4 weeks, 3 months, 6 months, and 12 months post-intervention. The baseline, 6-month, and 12-month follow-up assessments are conducted face-to-face, while the other two assessments are conducted over telephone. Because of the specific questions related to each intervention modality, volunteer callers cannot be blinded to the intervention modality, but they will be blinded to the study hypotheses. The intervention duration for the Tele-BA, Tele-MF, and Tele-BF groups is 4 weeks, with two sessions per week of approximately 30 minutes each (i.e., eight sessions in total). All intervention sessions are conducted by trained volunteers via telephone.

Randomization

After completing baseline assessment, participants are randomly assigned to either Tele-BA, Tele-MF, or Tele-BF using an online random number generated by a research assistant. Participants are then informed of their assigned intervention, and Tele-BA participants receive copies of BA worksheets. Due to the nature of the intervention, participants cannot be blinded as the types of activities in each intervention group are distinct.

Volunteer Training and Training Materials

Tele-BA intervention was adapted from the Telehealth Behavioral Activation Treatment Manual for Homebound Older Adults (55). The Tele-MF intervention was adapted from the smartphone-delivered mindfulness intervention developed by Lindsay et al. (2019) to reduce loneliness in stressed adults (20). The Tele-BF active control condition was adapted from the BEFRIENDAS: Befrienders Information Guide by the National Ageing Research Institute (2020) (51). Each intervention includes eight sessions, with two sessions per week of approximately 30 minutes each, summing up to a four-week intervention period in total.

One experienced social worker and one research assistant are hired to deliver training sessions for Tele-BA, Tele-MF, and Tele-BF volunteers separately, making sure that the volunteers adhere to the respective intervention manual developed by the research team. Each volunteer is required to attend 6 weekly training sessions (2 hours per week) before they can independently conduct the intervention with older adults. A small group format, with four to six volunteers, is used to include role plays and practice in each session. The research

team worked closely with the two trainers to prepare PowerPoint presentations to ensure comparability of the training content across small groups within the same intervention program.

Intervention Procedures and Delivery

Experimental Condition 1: Tele-BA

BA intervention covers a set of strategies to improve older adults' mood by (a) learning skills to help change daily behaviors, (b) cultivating new healthy behaviors, and (c) identifying and changing patterns of avoidance, withdrawal, and inactivity.

The first session provides an introduction to BA and describes the worksheet to record participants' daily activities. The second session focuses on behavioral monitoring and identification of important and meaningful life domains and goals. In the third session, participants are guided to select and plan the most rewarding activities to accomplish their chosen goals, and it is explained how to seek support for difficult activities. In the fourth session, participants are supported to identify and overcome barriers to implementing their selected activities. In the fifth session, participants are encouraged to find ways to maintain the benefits after the intervention program. In the sixth session, participants' behavioral monitoring and activity planning for the coming week is reinforced. In the seventh session, problem-solving and maintaining the benefits obtained after the end of the program are discussed. In the last session, participants' learning goals are reviewed, their successes are celebrated, and continued BA practice in daily life is discussed.

BA volunteers play an active role in the intervention to change participants' negative cognitive processes. In particular, they guide older adults to develop skills to manage their isolation and loneliness, cultivate confidence in their ability to do more than they think they can, and encourage them to take part in meaningful and pleasant activities.

Experimental Condition 2: Tele-MF

Tele-MF intervention is based on the smartphone-delivered mindfulness intervention developed by Lindsay et al. (2019) to reduce loneliness in stressed adults (20). The intervention aims to reduce loneliness and increase social interaction by cultivating two mindfulness skills: mindfulness orientation and present monitoring. To standardize Tele-MF intervention condition with the other two conditions (Tele-BA and Tele-BF), we modified the original approach of Lindsay et al. (2019) from 14 sessions of 20 minutes each (delivered over a period of 8 weeks) to 8 sessions of 30 minutes each (delivered over a period of 4 weeks) and from a smartphone app to a telephone-based intervention. Thus, eight telephone sessions are delivered to each participant.

In the first session, the concept of mindfulness is introduced. In the second session, body focus techniques with closed and open eyes are practiced. In the third session, participants learn how to focus on their body when listening to others talk. In the fourth session, participants learn how to focus on their body and mind using the verbal and mental labels of "feel" (i.e. the entire range of experiences in one's body, including affective states, body sensations, energy flow sensations, etc). In the fifth session, participants learn to feel the experiences

of their body while maintaining equanimity by keeping their body relaxed. In the sixth session, participants learn to distinguish different types of sensations, levels of intensity, and spatial patterns and to detect subtle body events. In the seventh session, participants practice to deepen equanimity by maintaining a positive attitude toward stress. In the final eighth session, the content of the training is summarized in an appreciative way and ways to continue MF practice are discussed.

Active Control Condition: Tele-BF

BF refers to regular contact between a volunteer who acts as or becomes friends with older adults. BF consists of a series of conversations between the participant and the volunteer, which are often related to everyday topics and events, such as hobbies, TV programs, cooking, books, or movies (56). Because BF aims at increasing communication and providing emotional support, but not other psychological processes, it has been widely used as the active control group in interventions on reducing loneliness (51). In this proposed study, a total of eight BF sessions for four weeks, each session lasting approximately 30 minutes.

The first session includes an introduction to BF and a self-introduction of the participant and the volunteer. For the second to eighth sessions the following specific conversation topics are suggested: second session, favorite TV programs; third session, diets, cooking, and favorite foods; fourth session, current news events or a memorable recent event; fifth session, weather and favorite season; sixth session, favorite soup; seventh session, favorite holiday and celebration activities; and eight session, favorite places and conclusion.

Materials and Measures

Structured questionnaires are used to assess participants at baseline and 4 weeks, 3 months, 6 months, and 12 months post-intervention. Table 1 shows an overview of timeline and measures in this study.

Primary Outcomes

Table 1 shows an overview of timeline and measures. Loneliness and perceived social support are the primary outcomes of the intervention.

Loneliness. Loneliness is measured by the De Jong Gierveld Loneliness Scale (56) and the 20-item Revised UCLA Loneliness Scale (58). The validated Chinese version of the De Jong Gierveld Loneliness Scale (Leung et al., 2008), consisting of six items, is adopted to measure retrospective loneliness. Participants are asked to indicate their level of agreement with the six statements on a 3-point scale (1 = yes, 2 = more or less, 3 = no). Sample items include “I experience a general sense of emptiness,” “Often, I feel rejected,” and “I miss having people around.” Means and standard deviations will be used in data analysis. The statements will be reversely scored with higher values representing higher loneliness.

The Revised UCLA Loneliness Scale (58) comprises 20 items to assess loneliness and has been validated among Chinese adolescents (59). This version improved from the original UCLA Loneliness Scale (60) by addressing concerns on the direction of the items, discriminant validity, social desirability (58). Each

item in the Revised UCLA Scale is a question starting with “How often do you feel?” Participants are asked to indicate how often they think each question describes their experiences on a 4-point scale (1 = never to 4 = always). Sample items include “How often do you feel alone?”; “How often do you feel that there is no one you can turn to?”; and “How often do you feel that you are no longer close to anyone?”. Item are averaged with higher score indicating higher perceptions of loneliness.

Perceived Social Support. Perceived social support is assessed using the Chinese version of the 12-item Multidimensional Scale of Perceived Social Support (MSPSS) (61,62). The MSPSS asks participants to indicate their level of agreement with 12 items on a 7-point scale (1 = strongly disagree to 7 = strongly agree). Sample items include “I get the help and support I need from my friends,” “I get the emotional help and support I need from my family,” and “My family is willing to help me make decisions.” Answers on all 12 items will be averaged to derive a total score of perceived social support, with higher values representing higher perceived social support.

Secondary Outcome Measures

The secondary outcomes include social network size, psychological well-being, life satisfaction, stress level, sleep quality, symptoms of depression and anxiety, physical health, and cognitive health.

Social network size. Social network size is assessed using the Chinese validated Lubben Social Network Scale (57,63). The Lubben Social Network Scale consists of two sets of three questions. The first set evaluates the kinship ties of participants: “How many relatives do you see or hear from at least once a month?”; “How many relatives do you feel close to, such that you could call them for help?”; and “How many relatives do you feel at ease with that you can talk about private matters?” The second set evaluates the non-kin ties of participants by asking three parallel questions. The only difference is that the word *relatives* is replaced with the word *friends*. Participants indicate the number of social partners on a 6-point scale (0 = none to 5 = more than 9 people), deriving in sum scores representing larger network sizes for a total of relatives/friends as well as for friends and family separately.

Psychological Well-being. Participants' psychological well-being is assessed using the Psychological Well-being Scale, originally developed by Ryff and colleagues (64). In this study, we utilize the validated brief version of the scale specifically tailored for the Hong Kong Chinese population, as validated by Chan, Chan, and Sun in 2017 (65). The scale consists of six dimensions of psychological functioning including autonomy (e.g., “have confidence in one’s opinions”), environmental mastery (e.g., No difficulty in arranging life”), personal growth (e.g., “Have developed a lot as a person”), positive relations with others (e.g., “Enjoy conversations with friends”), purpose in life (e.g., “Enjoy making plans for the future”), and self-acceptance (e.g., “Feel good about self”). Participants will be asked to rate to what extent they agree with each of the statement on a 6-point scale (1= strongly disagree to 6 = strongly agree).

Life Satisfaction. The Satisfaction with Life Scale, originally developed by Diener et al. (66) and validated in Chinese by Sachs (67), is utilized to assess participants' life satisfaction. Participants will be requested to indicate their agreement level with each of the five statements on a 7-point scale, ranging from 1 (strongly disagree) to 7 (strongly agree).

Stress Level. Stress level is measured using the 14-item Perceived Stress Scale in its validated Chinese translation (68,69). Participants indicate how often they have experienced the feelings described in the statements in the past month on a 5-point scale (0 = never to 4 = very often). Sample items include “In the last month, how often have you been upset because of something that happened unexpectedly?”; “In the last month, how often have you felt confident about your ability to handle your personal problems?”; and “In the last month, how often have you been able to control irritations in your life?”. As five items are reversed scored, they will be recoded before computing a mean score, with higher scores representing more stress.

Sleep Quality. Sleep quality is measured using the Chinese version of the eight-item Sleep Condition Indicator (70,71). Participants are asked to report their sleep quality and continuity, duration and frequency of insomnia symptoms, satisfaction with sleep, and frequency of sleep during the day on a 5-point scale from 0 to 4, with a higher score indicating better sleep quality. Mean scores will be calculated, with a higher score indicating better sleep quality.

Depressive Symptoms. Depressive symptoms are assessed using the nine-item Patient Health Questionnaire-9 in its validated Chinese version (PHQ-9) (72, 73). Participants are asked to report the frequency of cognitive, physical, and affective symptoms of depression in the past 2 weeks on a 4-point scale (0 = not at all to 4 = nearly every day). Sample items include “having little interest or pleasure in doing things,” “feeling tired or having low energy,” and “feeling down, depressed, or hopeless.” Means and standard deviations of the items will be used in data analysis, with a higher score indicating a higher level of depression.

Symptoms of Anxiety and Depression. The seven-item Hospital Anxiety and Depression Scale (HADS) is used to measure symptoms of anxiety and depression (Anxiety) (74). We adopted the Chinese validated version in Hong Kong (75). Participants indicate the intensity of each symptom on a 4-point scale (1 = not at all, 2 = several days, 3 = more than half the days, and 4 = nearly every day). Sample items include “feeling nervous, anxious, or on edge,” “not being able to stop or control worrying,” and “having trouble relaxing.” Means of the items will be used in data analysis, with a higher score indicating higher levels of anxiety and depression.

Physical Health. The physical health of older adults is assessed in four ways. First, self-rated health is measured by asking participants to rate their perceived general health, vision, and hearing status on a 5-point scale (1 = excellent to 5 = bad) (76). Reversed scores will be used in data analysis, with a higher score indicating better physical health.

Second, functional health is measured using the Chinese version of the WHO Disability Assessment Schedule 2.0 (WHODAS 2.0) (77,78). The WHODAS 2.0 consists of 36 items covering six domains (i.e.,

cognition, mobility, self-care, getting along, life activities, and participation). For each item, the participants are asked to rate their level of difficulty over the past 30 days in the domains of cognition (e.g., "Concentrating on doing something for 10 minutes"), mobility (e.g., "Standing for long periods such as 30 minutes"), self-care (e.g., "Washing your whole body"), getting along (e.g., "Dealing with people you do not know"), and life activities (e.g., "Taking care of your household responsibilities"). They are asked to rate the level of difficulty of activities in the domain of participation (e.g., "participating in community activities") on a 5-point scale (0 = none, 4 = extremely). Sum scores of each category will be used in data analysis, with a higher score indicating a greater level of difficulty.

In addition, chronic diseases are measured by asking participants to complete a list of 22 diseases using 1 for "yes" or 0 for "no" (79). Total score of the chronic diseases will be calculated.

Cognitive Health. The Hong Kong version of the Montreal Cognitive Assessment (MoCA) (80,81) is used to assess cognitive health. The MoCA has a possible total score of 30. To facilitate assessment over the phone, the full version of the MoCA is used at baseline, 6-month and 12-month follow-up, and the MoCA 5-minute protocol is used at the 4-week and 3-month follow-up assessments. The full version of the MoCA covers seven cognitive domains: executive functions, naming, attention, language, abstraction, delayed recall, and orientation. The MoCA 5-min protocol consists of four subtests covering five cognitive domains: attention, executive functions, language, orientation, and memory (81). Total scores will be used in data analysis, with a higher score indicating a greater level of cognitive performance.

Mediators

Mindfulness. Mindfulness is measured using the Five Facet Mindfulness Questionnaire in its Chinese version (FFMQ) (82, 83). The FFMQ measures an individual's general tendency to be mindful in their daily life. The scale contains 20 items covering five facets of mindfulness: observing, describing, acting with awareness, nonjudging of inner experience, and nonreactivity to inner experience. Participants are asked to rate to what extent the statements describe their thoughts and feeling on a 5-point scale (1 = never to 5 = always). Sample items include "I notice the smells and aromas of things (Observing)," "I am good at finding words to describe my feelings (Describing)," and "I perceive my feelings and emotions without having to react to them (Nonreactivity to inner experience). Means will be used in data analysis, with a higher score indicating a greater level of mindfulness.

Meaningful/Happy Events. Participants are asked to report up to three of the happiest and/or meaningful events in daily life over the past week. They first select the category of the event on a list of six options (i.e., interpersonal, recreation, spiritual, daily responsibility, learning, and other). They are then asked to rate the levels of happiness and meaningfulness of the event on a 5-point scale (1 = very low to 5 = very high). Means and standard deviations will be used in data analysis, with a higher score indicating a greater level of happiness or meaningfulness.

Biomarkers. One third of participants in the sample will be invited to provide saliva samples. After providing detailed guidelines in saliva sample collection, three saliva samples are collected by the participants using Salivette tubes at the participants' homes on 2 consecutive weekdays, three times each day: after waking up, before lunch, and before bedtime, 4 time points (baseline, 4-week, 3-month and 6-month follow-up). Standard instruction procedures and a telephone reminder are provided to participants. The collected Salivette tubes are kept frozen, and the cortisol levels are determined according to the manufacturer's standard using an ELISA kit (Salimetrics, PA, USA). Each participant's diurnal cortisol pattern is identified using four indicators: (1) morning cortisol, (2) evening cortisol, (3) mean cortisol, and (4) the diurnal cortisol slope. Salivary samples are also tested for CRP using the Salivary C-Reactive Protein ELISA kit according to the manufacturer's recommended protocol (Salimetrics, PA, USA).

Genotyping. DNA is collected from saliva samples at baseline, and the Infinium OmniZhongHua-8 v1.3 BeadChip with 887,270 SNPs are used, as this type of chip has been widely used for genotyping in Chinese populations. This type of chip is designed to pay particular attention to alleles found in Chinese populations and is the best choice in terms of study design and cost. It covers common, intermediate, and rare variants found in Chinese populations for genome-wide association studies (GWAS). According to the manufacturer's information, it covers 77% of common variants (minor allele frequency [MAF] > 5%), 73% of intermediate variants (MAF > 2.5%), and 65% of rare variants (MAF > 1%) in Chinese populations at $r^2 \geq 0.8$. The assay has exceptionally high data quality in terms of call rates (average > 99%), reproducibility (> 99.9%), and low sample repeat rates, and has high signal-to-noise ratios and low overall noise levels, enabling accurate and reliable calls and copy number analysis. The Sequenom platform is used in the second stage of variant genotyping. The Sequenom system is based on single-base extension (SBE) chemistry and mass spectroscopy, and allows SNPs to be designed for 40-plex assays. If it is necessary to sequence candidate genes, mutation screening is conducted by direct sequencing of exons, intron–exon boundaries, and conserved promoter elements using the ABI 3730XL sequencer in the CGS HKU genotyping core facility. Thus, candidate genes, GWAS, polygenic risk scores, and SNPs from underlying biological pathways are used as genetic dispositions.

Process Evaluation

To ensure the quality of and satisfaction with Tele-BA, Tele-MF, or Tele-BF intervention sessions, participants will be asked to complete the Treatment Evaluation Inventory (TEI) (84) and two additional questions about their volunteer's delivery of interventions at their 4-week follow-up assessment.

The 11-item TEI is forward-translated into Chinese and backward-translated according to Brislin (1980) (85) and then adapted to the context of interventions on reducing loneliness. Participants will be asked to rate the acceptability (e.g., "I find this treatment to be an acceptable way of dealing with loneliness"), appropriateness (e.g., "I find this treatment to be an appropriate way of dealing with loneliness"), and predicted effectiveness (e.g., "I believe this treatment is likely to be effective") of the interventions they received on a 7-

point scale (1 = not at all to 7 = very much). The mean of the items will be used in data analysis, with higher scores indicating more favorable evaluation.

In addition to questions about the intervention, participants will be asked to rate their satisfaction with their volunteer. They will be asked to rate the extent to which they perceive their volunteer as an effective interventionist on a 7-point scale (1 = not at all to 7 = very much). Means will be used in data analysis, with a higher score indicating greater satisfaction.

Demographic Information

In addition, the following sociodemographic information is collected as potential covariates: (1) age; (2) gender; (3) marital status; (4) number of children; (5) education level; (6) employment status; (7) household income.

Planned Statistical Analysis of the Effects of the Intervention

Planned Preliminary Analysis

Descriptive statistics, correlation analysis, and attrition analysis (t-tests to compare the covariates and outcome variables of participants who drop out and those who complete the intervention) and randomization tests (one-way analysis of variance [ANOVA]) are conducted to test for differences in covariates and outcome variables between conditions using SPSS 28.

Planned Analysis of the Effects of the Intervention

The effects of the intervention on loneliness are assessed using mixed models for repeated measures. Specifically, a conditional growth model analysis with an unstructured covariance matrix is conducted using a Group (two intervention groups and one control group) × Time (baseline, 4 weeks, and 3-, 6-, and 12-month follow-ups) interaction as an indicator of the intervention effects. For sensitivity analysis, a similar mixed model for repeated measures with an unstructured covariance matrix, but with the ANOVA model, is used. The demographic characteristics that show significant differences across groups at baseline are controlled for in the models. In addition, an "intention-to-treat" analysis is used to assess differences in outcomes by incorporating participants who do not complete the study. The intention-to-treat principle is also applied, and all statistical analyses are conducted using SPSS 28. In order to test the effect of the proposed mediators, path analyses with manifest variables will be run with indirect effect specified and tested with 95% bias-corrected confidence intervals resulting from 1000 bootstraps in Mplus 8. Mindfulness, happy/meaningful events, CRP, the position of candidate genes, polygenic risk scores and SNPs are potential mediators to be tested.

Discussion

Participant recruitment of the study started in 20 April 2022 and completed in 30 April 2023. The project is under the stage of data collection and we estimated to complete data collection in April 2024. The protocol paper has been completed and submitted on 4 April 2023 before completing participant recruitment. However, due to technical problems encountered in submission, the submission of the protocol was delayed.

Supplement 1. Study Protocol

Although there are a number of interventions that have successfully reduced loneliness in the literature, few focus on older adults as their target despite the fact that a large proportion of older adults report loneliness (1,52). One challenge in delivering any intervention to lonely older adults is that it is difficult to approach them because of their low mobility and sociality. Such challenges become particularly conspicuous during the pandemic. In view of this, we propose the study described in this article to investigate the effectiveness of two telephone-based interventions compared to an active control.

This trial will be the first to test telephone-based interventions in reducing loneliness in older adults in Hong Kong. We will compare the effectiveness of an approach aiming to increase positive and meaningful experiences (i.e., behavioral activation) and an approach aiming to decrease negative experiences (i.e., mindfulness meditation) against an active control group in reducing loneliness. In addition to testing the main effects of the interventions, we will test the behavioral and biological mechanisms underlying the effects.

Telephone-based interventions enable delivery of interventions to people who are difficult to reach because of mobility and/or travel constraints. Such method is particularly beneficial to low-income older adults who live alone and have limited/no internet access during the pandemic. If the method is shown to be successful in Hong Kong, it will facilitate the launch of large-scale community-based interventions in the future.

To summarize, if the interventions are found to be effective, they will provide a scientific understanding on the effectiveness of the two intervention approaches to reduce loneliness. The findings will facilitate the establishment of large-scale interventions to reduce loneliness and promote improved well-being among older adults in Hong Kong.

Trial Status

The current protocol is version 2, 29 September 2023 with the first version completed on 4 April 2023. Recruitment began on the 20st April 2021 and participant recruitment was completed on 30 April 2023. Follow-up of participants will continue until April/May 2024.

List of abbreviations

BA: Behavioral activation
MF: Mindfulness
BF: Befriending
MBSR: Mindfulness-Based Stress Reduction
CRP: C-reactive protein
MAT: Monitor and Acceptance Theory
RCT: Randomized Controlled Trial
Tele-BA: telephone-delivered behavioral activation
Tele-MF: telephone-delivered Mindfulness
Tele-BF: Tele-Befriending

MoCA: Montreal Cognitive Assessment
FFMQ: Five Facet Mindfulness Questionnaire
SBE: single-base extension
TEI: Treatment Evaluation Inventory
ANOVA: one-way analysis of variance
SPIRIT: Standard Protocol Items—Recommendations for Interventional Trials
CONSORT: Consolidated Standards of Reporting Trials

Supplement 1. Study Protocol

Table 1. SPIRIT schedule of enrolment, interventions, and assessments.

	STUDY PERIOD									
	Enrol-ment	Allo-cation	Post-allocation							
			Intervention period							
TIMEPOINT	*-1*	Base-line (T0)	Week 1	Week 2	Week 3	Week 4	*4-week follow-up post Intervention (T1)*	*3-month follow-up post Intervention (T2)*	*6-month follow-up post Intervention (T3)*	*12-month follow-up post Intervention (T4)*
ENROLMENT:										
Eligibility screen	X									
Informed consent	X									
Allocation		X								
INTERVENTIONS:										
Tele-BA			X	X	X	X				
Tele-MF			X	X	X	X				
Tele-BF			X	X	X	X				
ASSESSMENTS:										
Socio-demographic variables		X								
Covariates		X					X			
Primary Outcome		X					X	X	X	X
Secondary Outcomes		X					X	X	X	X
Mediator variables		X					X	X	X	X

Declarations

Ethics approval and consent to participate

Ethical approval for this RCT has been obtained by the Human Research Ethics Committee (HREC) of the Hong Kong University of Education (Reference Number: 2019-2020-0442). Consent to participate will be obtained from each participant. The study was prospectively registered in the Clinical Trials Registry of the University of Hong Kong Clinical Trials Centre (HKU-CTC) (registration number: HKUCTR-2929) and retrospectively registered at the Chinese Clinical Trial Registry in the WHO Registry Network, number ChiCTR2300072909). The contents of the two registrations were identical.

Consent for publication

Yes, the consent has been received from participants.

Availability of data and material

The datasets generated during the study will be available from the corresponding author on reasonable request.

Competing interests

The authors declare that they have no competing interests.

Funding

The RCT is funded by the Research Grants Council of the Hong Kong Special Administrative Region Government (project number: C8105-20GF), Research Grant Council of the Hong Kong Special Administrative Region Government.

Authors' contributions

KLC applied for funding for the RCT. NGC developed the original versions of the Behavioral Activation and Befriending interventions. DY coordinated on translating all intervention materials into Chinese. DY and JYYK adapted the translated the intervention material into the context of Cantonese. DJ, DY, and KLC planned and conduct the RCT on Hong Kong. DJ and DY wrote up the first draft of this protocol. DJ, LMW, NGC, and JYYK revised this draft considerably. RTHH and YS contributed to develop contents related to biomarkers and genotyping, respectively. All authors read, commented and approved the final manuscript.

Acknowledgements

The research team would like to acknowledge all the participants, for their support and participation, in the study. The authors thank Gloria Jiahui Lin, Sophy Shuang Liu, Hollie Ho Yeng Mo, and Daisy Shuting Li for their research assistance.

References

1. Ong AD, Uchino BN, Wethington E. Loneliness and health in older adults: A mini-review and synthesis. Gerontology. 2016;62(4):443–9.
2. Yang F, Gu D. Widowhood, widowhood duration, and loneliness among older adults in China. Soc Sci Med. 2021;283:114179.
3. Yang K, Victor C. Age and loneliness in 25 European nations. Ageing Soc. 2011;31(8):1368–88.
4. Luhmann M, Hawkley LC. Age differences in loneliness from late adolescence to oldest old age. Dev Psychol. 2016;52(6):943.
5. Cacioppo JT, Hawkley LC, Ernst JM, Burleson M, Berntson GG, Nouriani B, et al. Loneliness within a nomological net: An evolutionary perspective. J Res Personal. 2006;40(6):1054–85.
6. Lutzman M, Sommerfeld E, Ben-David S. Loneliness and social integration as mediators between physical pain and suicidal ideation among elderly men. Int Psychogeriatr. 2021;33(5):453–9.
7. Kotwal AA, Holt-Lunstad J, Newmark RL, Cenzer I, Smith AK, Covinsky KE, et al. Social isolation and loneliness among San Francisco Bay Area older adults during the COVID-19 shelter-in-place orders. J Am Geriatr Soc. 2021;69(1):20–9.
8. Stolz E, Mayerl H, Freidl W. The impact of COVID-19 restriction measures on loneliness among older adults in Austria. Eur J Public Health. 2021;31(1):44–9.
9. Karagöz D, Ramkissoon H. Loneliness, travel nostalgia, subjective well-being and prevention regulatory focus: a moderated mediation model analysis. Curr Issues Tour. 2023;1–17.
10. Lee EE, Depp C, Palmer BW, Glorioso D, Daly R, Liu J, et al. High prevalence and adverse health effects of loneliness in community-dwelling adults across the lifespan: role of wisdom as a protective factor. Int Psychogeriatr. 2019;31(10):1447–62.
11. Lucas GM, Knowles ML, Gardner WL, Molden DC, Jefferis VE. Increasing social engagement among lonely individuals: The role of acceptance cues and promotion motivations. Pers Soc Psychol Bull. 2010;36(10):1346–59.
12. Higgins ET. Self-discrepancy: a theory relating self and affect. Psychol Rev. 1987;94(3):319.
13. Russell JA, Carroll JM. On the bipolarity of positive and negative affect. Psychol Bull. 1999;125(1):3.
14. Kessler EM, Staudinger UM. Affective experience in adulthood and old age: The role of affective arousal and perceived affect regulation. Psychol Aging. 2009;24(2):349.
15. Kuhl J. A functional-design approach to motivation and self-regulation: The dynamics of personality systems interactions. In: Handbook of self-regulation. Elsevier; 2000. p. 111–69.
16. Coll-Planas L, del Valle Gomez G, Bonilla P, Masat T, Puig T, Monteserin R. Promoting social capital to alleviate loneliness and improve health among older people in S pain. Health Soc Care Community. 2017;25(1):145–57.

17. Choi NG, Caamano J, Vences K, Marti CN, Kunik ME. Acceptability and effects of tele-delivered behavioral activation for depression in low-income homebound older adults: in their own words. Aging Ment Health. 2021;25(10):1803–10.
18. Meeks S, Looney SW, Van Haitsma K, Teri L. BE-ACTIV: a staff-assisted behavioral intervention for depression in nursing homes. The Gerontologist. 2008;48(1):105–14.
19. Creswell JD, Irwin MR, Burklund LJ, Lieberman MD, Arevalo JMG, Ma J, et al. Mindfulness-Based Stress Reduction training reduces loneliness and pro-inflammatory gene expression in older adults: A small randomized controlled trial. Brain Behav Immun. 2012;26(7):1095–101.
20. Lindsay EK, Young S, Brown KW, Smyth JM, Creswell JD. Mindfulness training reduces loneliness and increases social contact in a randomized controlled trial. Proc Natl Acad Sci. 2019;116(9):3488–93.
21. Cacioppo S, Grippo AJ, London S, Goossens L, Cacioppo JT. Loneliness: Clinical import and interventions. Perspect Psychol Sci. 2015;10(2):238–49.
22. Masi CM, Chen HY, Hawkley LC, Cacioppo JT. A meta-analysis of interventions to reduce loneliness. Personal Soc Psychol Rev. 2011;15(3):219–66.
23. Dimidjian S, Barrera Jr M, Martell C, Muñoz RF, Lewinsohn PM. The origins and current status of behavioral activation treatments for depression. Annu Rev Clin Psychol. 2011;7:1–38.
24. Ferster CB. A functional analysis of depression. Am Psychol. 1973;28(10):857.
25. Lejuez CW, Hopko DR, Acierno R, Daughters SB, Pagoto SL. Ten year revision of the brief behavioral activation treatment for depression: revised treatment manual. Behav Modif. 2011;35(2):111–61.
26. Orgeta V, Brede J, Livingston G. Behavioural activation for depression in older people: systematic review and meta-analysis. Br J Psychiatry. 2017;211(5):274–9.
27. Martell CR, Kanter J. Behavioral activation in the context of "third wave" therapies. Accept Mindfulness Cogn Behav Ther Underst Appl New Ther. 2011;193–209.
28. Richards DA, Ekers D, McMillan D, Taylor RS, Byford S, Warren FC, et al. Cost and Outcome of Behavioural Activation versus Cognitive Behavioural Therapy for Depression (COBRA): a randomised, controlled, non-inferiority trial. The Lancet. 2016;388(10047):871–80.
29. Soucy I, Provencher MD, Fortier M, McFadden T. Secondary outcomes of the guided self-help behavioral activation and physical activity for depression trial. J Ment Health. 2019;28(4):410–8.
30. Käll A, Jägholm S, Hesser H, Andersson F, Mathaldi A, Norkvist BT, et al. Internet-based cognitive behavior therapy for loneliness: a pilot randomized controlled trial. Behav Ther. 2020;51(1):54–68.
31. Choi NG, Pepin R, Marti CN, Stevens CJ, Bruce ML. Improving social connectedness for homebound older adults: randomized controlled trial of tele-delivered behavioral activation versus tele-delivered friendly visits. Am J Geriatr Psychiatry. 2020;28(7):698–708.

32. Burke L, Littlewood E, Gascoyne S, McMillan D, Chew-Graham CA, Bailey D, et al. Behavioural Activation for Social IsoLation (BASIL+) trial (Behavioural activation to mitigate depression and loneliness among older people with long-term conditions): Protocol for a fully-powered pragmatic randomised controlled trial. PLoS One. 2022;17(3):e0263856.
33. Pellas J, Renner F, Ji JL, Damberg M. Telephone-based behavioral activation with mental imagery for depression: A pilot randomized clinical trial in isolated older adults during the Covid-19 pandemic. Int J Geriatr Psychiatry. 2022;37(1).
34. Chodron P. When things fall apart: Heart advice for difficult times. Shambhala Publications; 2000.
35. Barnes S, Brown KW, Krusemark E, Campbell WK, Rogge RD. The role of mindfulness in romantic relationship satisfaction and responses to relationship stress. J Marital Fam Ther. 2007;33(4):482–500.
36. Kwok JYY, Choi EPH, Lee JJ, Lok KYW, Kwan JCY, Mok VCT, et al. Effects of Mindfulness Yoga Versus Conventional Physical Exercises on Symptom Experiences and Health-related Quality of Life in People with Parkinson's Disease: The Potential Mediating Roles of Anxiety and Depression. Ann Behav Med. 2022 Oct 3;56(10):1068–81.
37. Berry DR, Cairo AH, Goodman RJ, Quaglia JT, Green JD, Brown KW. Mindfulness increases prosocial responses toward ostracized strangers through empathic concern. J Exp Psychol Gen. 2018;147(1):93.
38. Carson JW, Carson KM, Gil KM, Baucom DH. Mindfulness-based relationship enhancement. Behav Ther. 2004;35(3):471–94.
39. Kwok JYY, Kwan JCY, Auyeung M, Mok VCT, Lau CKY, Choi KC, et al. Effects of mindfulness yoga vs stretching and resistance training exercises on anxiety and depression for people with Parkinson disease: A randomized clinical trial. JAMA Neurol. 2019;76(7):755–63.
40. Davis MC, Zautra AJ. An online mindfulness intervention targeting socioemotional regulation in fibromyalgia: results of a randomized controlled trial. Ann Behav Med. 2013;46(3):273–84.
41. Geiger PJ, Boggero IA, Brake CA, Caldera CA, Combs HL, Peters JR, et al. Mindfulness-based interventions for older adults: A review of the effects on physical and emotional well-being. Mindfulness. 2016;7:296–307.
42. Lindsay EK, Creswell JD. Mechanisms of mindfulness training: Monitor and Acceptance Theory (MAT). Clin Psychol Rev. 2017 Feb;51:48–59.
43. Wister A, Eireann O, Fyffe I, Cosco TD. Technological interventions to reduce loneliness and social isolation among community-living older adults: A scoping review. Gerontechnology. 2021;20(2):1–16.
44. Tsai HH, Cheng CY, Shieh WY, Chang YC. Effects of a smartphone-based videoconferencing program for older nursing home residents on depression, loneliness, and quality of life: a quasi-experimental study. BMC Geriatr. 2020;20(1):27.

45. Kim JW, Stewart R, Kang SJ, Jung SI, Kim SW, Kim JM. Telephone based interventions for psychological problems in hospital isolated patients with COVID-19. Clin Psychopharmacol Neurosci. 2020;18(4):616.
46. Anvari MS, Kleinman MB, Dean D, Bradley VD, Abidogun TM, Hines AC, et al. Adapting a Behavioral Activation Intervention for Opioid Use Disorder and Methadone Treatment Retention for Peer Delivery in a Low-Resource Setting: A Case Series. Cogn Behav Pract. 2023;
47. Raue PJ, Sirey JA, Dawson A, Berman J, Bruce ML. Lay-delivered behavioral activation for depressed senior center clients: Pilot RCT. Int J Geriatr Psychiatry. 2019;34(11):1715–23.
48. Renn BN, Sams N, Areán PA, Raue PJ. A low-intensity behavioral intervention for depression in older adults delivered by lay coaches: proof-of-concept trial. Aging Ment Health. 2022;1–8.
49. Warner LM, Jiang D, Yeung DY, Choi N, Ho RTH, Kwok JYY, Song Y, Chou KL. Study protocol of an RCT testing the effects of volunteering on loneliness, perceived support, well-being and cognitive functioning in older adults. (under review).
50. Davidson S, Rossall P. Evidence review: Loneliness in later life. White Pap. 2015;1–29.
51. Doyle C, Bhar S, Fearn M, Ames D, Osborne D, You E, et al. The impact of telephone-delivered cognitive behaviour therapy and befriending on mood disorders in people with chronic obstructive pulmonary disease: A randomized controlled trial. Br J Health Psychol. 2017;22(3):542–56.
52. Cheung G, Wright-St Clair V, Chacko E, Barak Y. Financial difficulty and biopsychosocial predictors of loneliness: A cross-sectional study of community dwelling older adults. Arch Gerontol Geriatr. 2019;85:103935.
53. Yang F, Gu D. Predictors of loneliness incidence in Chinese older adults from a life course perspective: a national longitudinal study. Aging Ment Health. 2020;24(6):879–88.
54. Lin CY, Tsai CS, Fan CW, Griffiths MD, Chang CC, Yen CF, et al. Psychometric evaluation of three versions of the UCLA Loneliness Scale (full, eight-item, and three-item versions) among sexual minority men in Taiwan. Int J Environ Res Public Health. 2022;19(13):8095.
55. Choi NG, DiNitto DM, Marti CN, Choi BY. Telehealth use among older adults during COVID-19: Associations with sociodemographic and health characteristics, technology device ownership, and technology learning. J Appl Gerontol. 2022;41(3):600–9.
56. Mead N, Lester H, Chew-Graham C, Gask L, Bower P. Effects of befriending on depressive symptoms and distress: systematic review and meta-analysis. Br J Psychiatry. 2010;196(2):96–101.
57. Leung GTY, de Jong Gierveld J, Lam LCW. Validation of the Chinese translation of the 6-item De Jong Gierveld Loneliness Scale in elderly Chinese. Int Psychogeriatr. 2008;20(6):1262.
58. Chou KL, Jun LW, Chi I. Assessing Chinese older adults' suicidal ideation: Chinese version of the Geriatric Suicide Ideation Scale. Aging Ment Health. 2005 Mar 1;9(2):167–71.

59. Russell D, Peplau LA, Cutrona CE. The revised UCLA Loneliness Scale: Concurrent and discriminant validity evidence. J Pers Soc Psychol. 1980;39(3):472–80.
60. Russell D, Peplau LA, Ferguson ML. Developing a measure of loneliness. J Pers Assess. 1978;42(3):290–4.
61. Chou KL. Assessing Chinese adolescents' social support: the multidimensional scale of perceived social support. Personal Individ Differ. 2000;28(2):299–307.
62. Zimet GD, Dahlem NW, Zimet SG, Farley GK. The multidimensional scale of perceived social support. J Pers Assess. 1988;52(1):30–41.
63. Gierveld JDJ, Tilburg TV. A 6-item scale for overall, emotional, and social loneliness: Confirmatory tests on survey data. Res Aging. 2006;28(5):582–98.
64. Ryff CD, Singer B. Psychological well-being: Meaning, measurement, and implications for psychotherapy research. Psychotherapy and psychosomatics. 1996 Feb 18;65(1):14-23.
65. Chan DW, Chan LK, Sun X. Developing a brief version of Ryff's scale to assess the psychological well-being of adolescents in Hong Kong. European Journal of Psychological Assessment. 2017 Apr 7.
66. Diener ED, Emmons RA, Larsen RJ, Griffin S. The satisfaction with life scale. Journal of personality assessment. 1985 Feb 1;49(1):71-5.
67. Sachs J. Validation of the satisfaction with life scale in a sample of Hong Kong university students. Psychologia. 2003;46(4):225-34.
68. Cohen S, Kamarck T, Mermelstein R. A Global Measure of Perceived Stress. J Health Soc Behav. 1983 Apr 11;24(4):385–96.
69. Leung DYP, Lam T hing, Chan SSC. Three versions of Perceived Stress Scale: validation in a sample of Chinese cardiac patients who smoke. BMC Public Health. 2010;10(1):513.
70. Espie CA, Kyle SD, Hames P, Gardani M, Fleming L, Cape J. The Sleep Condition Indicator: a clinical screening tool to evaluate insomnia disorder. BMJ Open. 2014;4(3).
71. Wong ML, Lau KNT, Espie CA, Luik AI, Kyle SD, Lau EYY. Psychometric properties of the Sleep Condition Indicator and Insomnia Severity Index in the evaluation of insomnia disorder. Sleep Med. 2017;33:76–81.
72. Spitzer RL, Kroenke K, Williams JBW, Group PHQPCS. Validation and utility of a self-report version of PRIME-MD: the PHQ primary care study. Jama. 1999;282(18):1737–44.
73. Yu X, Tam WWS, Wong PTK, Lam TH, Stewart SM. The Patient Health Questionnaire-9 for measuring depressive symptoms among the general population in Hong Kong. Compr Psychiatry. 2012;53(1):95–102.
74. Zigmond AS, Snaith RP. The hospital anxiety and depression scale. Acta Psychiatr Scand. 1983;67(6):361–70.

75. Leung CM, Wing YK, Kwong PK, Shum ALK. Validation of the Chinese-Cantonese version of the Hospital Anxiety and Depression Scale and comparison with the Hamilton Rating Scale of Depression. Acta Psychiatr Scand. 1999;100(6):456–61.
76. Shu X, Lin T, Wang H, Zhao Y, Jiang T, Peng X, Yue J. Diagnosis, prevalence, and mortality of sarcopenia in dialysis patients: a systematic review and meta-analysis. Journal of cachexia, sarcopenia and muscle. 2022 Feb;13(1):145-58.
77. Cheung MKT, Hung ATF, Poon PKK, Fong DYT, Li LSW, Chow ESL, et al. Validation of the World Health Organization Assessment Schedule II Chinese Traditional Version (WHODAS II CT) in persons with disabilities and chronic illnesses for Chinese population. Disabil Rehabil. 2015 Sep 25;37(20):1902–7.
78. Üstün TB, Chatterji S, Kostanjsek N, Rehm J, Kennedy C, Epping-Jordan J, et al. Developing the World Health Organization Disability Assessment Schedule 2.0. Bull World Health Organ. 2010 Nov 1;88(11):815–23.
79. Jiang D, Warner LM, Chong AML, Li T, Wolff JK, Chou KL. Promoting Volunteering Among Older Adults in Hong Kong: A Randomized Controlled Trial. The Gerontologist [Internet]. 2019 Jun 22; Available from: https://doi.org/10.1093/geront/gnz076
80. Nasreddine ZS, Phillips NA, Bédirian V, Charbonneau S, Whitehead V, Collin I, et al. The Montreal Cognitive Assessment, MoCA: a brief screening tool for mild cognitive impairment. J Am Geriatr Soc. 2005;53(4):695–9.
81. Wong A, Xiong YY, Kwan PWL, Chan AYY, Lam WWM, Wang K, et al. The validity, reliability and clinical utility of the Hong Kong Montreal Cognitive Assessment (HK-MoCA) in patients with cerebral small vessel disease. Dement Geriatr Cogn Disord. 2009;28(1):81–7.
82. Baer RA, Smith GT, Hopkins J, Krietemeyer J, Toney L. Using self-report assessment methods to explore facets of mindfulness. Assessment. 2006;13(1):27–45.
83. Hou J, Wong SYS, Lo HHM, Mak WWS, Ma HSW. Validation of a Chinese version of the Five Facet Mindfulness Questionnaire in Hong Kong and development of a short form. Assessment. 2014;21(3):363–71.
84. Kazdin AE. Acceptability of alternative treatments for deviant child behavior. J Appl Behav Anal. 1980;13(2):259–73.
85. Brislin RW. Cross-cultural research methods. In: Environment and culture. Springer; 1980. p. 47–82.
