# Supplementary material for: Behavioral Activation and Mindfulness Interventions in Reducing Loneliness and Improving Well-Being in Older Adults: The HEAL-HOA Randomized Clinical Trial
Source: JAMA Netw Open. 2026 Feb 4;9(2):e2557170. doi: 10.1001/jamanetworkopen.2025.57170 (PMC12873770; doi:10.1001/jamanetworkopen.2025.57170)
Supplement: Supplement 2. — eTable 1. Session Content of Tele-BA eTable 2. Session Content of Tele-MF eTable 3. Characteristics of Completers and Non-Completers at Baseline eTable 4. Mixed-Effects Analysis at 12 months (Sensitivity) eTable 5. Mixed-Effects Analysis at 12 months (Per-protocol) [file jamanetwopen-e2557170-s002.pdf]

Supplemental Online Content

Tang VFY, Jiang D, Kwok JYY, et al. Behavioral activation and mindfulness interventions in reducing loneliness and improving well-being in older adults: The HEAL-HOA randomized clinical trial. *JAMA Netw Open*. 2026;9(2):e2557170. doi:10.1001/jamanetworkopen.2025.57170

eTable 1. Session Content of Tele-BA
eTable 2. Session Content of Tele-MF
eTable 3. Characteristics of Completers and Non-Completers at Baseline
eTable 4. Mixed-Effects Analysis at 12 months (Sensitivity)
eTable 5. Mixed-Effects Analysis at 12 months (Per-protocol)

This supplemental material has been provided by the authors to give readers additional information about their work.

© 2026 Tang VFY et al. *JAMA Network Open.*

eTable 1. Session Content of Tele-BA

Session	Topic
1	Introduction to behavioral activation and record daily activity
2	Behavior monitoring, and identify important and meaningful life domains, values, and activities
3	Guide participants to understand what is important to them, and select and plan rewarding activities
4	Identify and overcome obstacles and seek for support for difficult activities
5	Guide participants to think about how to maintain gains after this intervention program
6	Reinforce behavior monitoring and activity planning in the coming week
7	Continue the discussion on problem solving and how to maintain gains after BA
8	Review BA learning goals and celebrate successes

© 2026 Tang VFY et al. *JAMA Network Open.*

eTable 2. Session Content of Tele-MF

Session	Topic
1	Introduction to mindfulness
2	Exercise on body focus techniques with closed and open eyes
3	Exercise on body focus techniques when listening to others talk
4	Exercise on body and mind focus techniques using verbal and mental labels of feel
5	Exercise on body focus techniques while maintaining equanimity by keeping their body relaxed
6	Exercise on distinguishing different types of sensations, levels of intensity, and spatial patterns and to detect subtle body events
7	Exercise on thoughts and alternative viewpoints and maintaining a positive attitude toward stress
8	Practice review, body scan, exercise on looking forward and preparing for the future

© 2026 Tang VFY et al. *JAMA Network Open.*

eTable 3. Characteristics of Completers and Non-Completers at Baseline

	**Completers (n=452)**	**Non-Completers (n=699)**
	n (%)	n (%)
Age, mean (SD), y	77.18 (0.30)	75.61 (0.34)
Gender		
Male	131 (29.0)	177 (25.3)
Female	132 (71.0)	522 (74.7)
Marital status		
Married	34 (7.5)	41 (5.9)
Divorced	135 (29.9)	194 (27.8)
Single (never married)	67 (14.8)	77 (11.0)
Widow	216 (47.8)	387 (55.4)
Education level		
No education	109 (24.1)	225 (32.2)
Primary or below	175 (38.7)	273 (39.1)
Secondary	148 (32.7)	178 (25.5)
Tertiary	20 (4.4)	23 (3.3)
Children		
Without children	116 (25.7)	172 (24.6)
With children	336 (74.3)	527 (75.4)
Chronic Illnesses[a]		
Did not have chronic illnesses	84 (18.6)	102 (14.6)
Had chronic illnesses	368 (81.4)	597 (85.4)
UCLA-LS, mean (SD)[b]	51.54 (0.28)	50.80 (0.33)
DJGL, mean (SD)[c]	4.65 (0.05)	4.44 (0.07)
PSS, mean (SD)[d]	17.86 (0.43)	14.50 (0.57)
MSPSS, mean (SD)[e]	37.93 (0.55)	40.24 (0.72)
SCI, mean (SD)[f]	22.69 (0.29)	21.90 (0.39)
SWLS, mean (SD)[g]	18.45 (0.15)	19.11 (0.17)
PWB, mean (SD)[h]	55.56 (0.29)	56.40 (0.36)
PHQ-9, mean (SD)[i]	2.35 (0.15)	2.50 (0.18)
HADS-A, mean (SD)[j]	2.21 (0.14)	2.23 (0.17)
LSNS, mean (SD)[k]	7.45 (0.21)	8.52 (0.29)

*Note*s.
Abbreviations: DJGL, De Jong Gierveld Loneliness Scale; HADS-A, Hospital Anxiety and Depression Scale – Anxiety Subscale; MSPSS, Multidimensional Scale of Perceived Social Support; PHQ-9, Patient Health Questionnaire – 9 item; PSS, Perceived Stress Scale; PWB, Psychological Well-Being Scale; SCI, Sleep Condition Indicator; SWLS, Satisfaction with Life Scale; UCLA-LS, UCLA Loneliness Scale;
[a]Chronic non-specific lung diseases, cardiac disease, peripheral disease, stroke, diabetes mellitus, arthritis, and cancer.
[b]UCLA-LS score range 20-80, with 20 indicating low level of loneliness and 80 indicating high level of loneliness.
[c]DJGL score range 0-6, with 0 indicating no loneliness and 6 indicating severe loneliness.
[d]PSS score range 0-56, with 0 indicating no perceived stress and 56 indicating extremely high perceived stress.
[e]MSPSS score range 12-84, with 12 indicating low perceived social support and 84 indicating high perceived social support.
[f]SCI score range 0-32, with 0 indicating poor sleep quality and 32 indicating good sleep quality.
[g]SWLS score range 5-35, with 5 indicating low life satisfaction and 35 indicating high life satisfaction.
[h]PWB score range 16-96, with 16 indicating low psychological well-being and 96 indicating high psychological well-being.
[i]PHQ-9 score range 0-27, with 0 indicating no depressive symptoms and 27 indicating severe depressive symptoms.
[j]HADS-A score range 0-21, with 0 indicating no anxiety and 21 indicating severe anxiety.
[k]LSNS score range 0-30, with 0 indicating severe social isolation and 30 indicating strong social integration.

© 2026 Tang VFY et al. *JAMA Network Open.*

eTable 4. Mixed-Effects Analysis at 12 months (Sensitivity)[a]

	Estimates across assessments, mean (95% CI) [b]						Tele-BA vs Tele-BF			Tele-MF vs Tele-BF			Overall between-group difference
Measures	Tele-BA (n=335)	*P* value	Tele-MF (n=460)	*P* value	Tele-BF (n=356)	*P* value	MD (95% CI)	Cohen d	MD *P* value	MD (95% CI)	Cohen d	MD *P* value	***P* value**
**Primary Outcomes**													
UCLA-LS[c]	47.86 (47.52, 48.19)	<.001	47.80 (47.52, 48.09)	<.001	48.62 (48.29, 48.94)	<.001	-0.76 (-1.33, -0.19)	0.11	0.004	-0.81 (-1.34, -0.29)	0.11	<.001	<.001
DJGL[d]	3.82 (3.76, 3.88)	<.001	3.92 (3.87, 3.97)	<.001	3.96 (3.90, 4.02)	<.001	-0.14 (-0.24, -0.04)	0.09	0.003	-0.04 (-0.14, 0.05)	0.07	0.77	<.001
**Secondary Outcomes**													
SCI[e]	22.33 (22.01, 22.65)	<.001	22.14 (21.87, 22.41)	<.001	21.86 (21.55, 22.17)	0.51	0.47 (-0.08, 1.02)	0.06	0.12	0.28 (-0.23, 0.79)	0.08	0.55	<.001
PWB[f]	60.32 (59.97, 60.66)	<.001	59.80 (59.51, 60.10)	<.001	59.81 (59.47, 60.14)	<.001	0.51 (-0.08, 1.10)	0.14	0.11	-0.005 (-0.55, 0.54)	0.14	>.99	<.001
MSPSS[g]	43.78 (43.15, 44.41)	<.001	43.81 (43.27, 44.34)	<.001	43.26 (42.65, 43.87)	<.001	0.52 (-0.55, 1.60)	0.11	0.74	0.55 (-0.44, 1.54)	0.17	0.54	<.001
PHQ-9[h]	2.48 (2.32, 2.63)	0.87	2.61 (2.48, 2.74)	0.02	2.72 (2.57, 2.87)	0.01	-0.24 (-0.50, 0.02)	0.34	0.07	-0.11 (-0.35, 0.13)	0.33	0.77	<.001
HADS-A[i]	2.12 (1.99, 2.26)	0.42	2.33 (2.22, 2.44)	0.01	2.29 (2.16, 2.42)	0.002	-0.17 (-0.39, 0.06)	0.33	0.23	0.04 (-0.17, 0.25)	0.30	>.99	<.001
PSS[j]	15.04 (14.65, 15.44)	<.001	15.66 (15.32, 16.01)	0.92	15.16 (14.77, 15.56)	<.001	-0.12 (-0.80, 0.56)	0.02	>.99	0.50 (-0.15, 1.15)	0.21	0.20	<.001
SWLS[k]	20.83 (20.66, 21.01)	<.001	20.67 (20.52, 20.82)	<.001	20.60 (20.43, 20.77)	<.001	0.24 (-0.06, 0.54)	0.13	0.17	0.08 (-0.20, 0.36)	0.15	1.000	<.001

Notes.

Abbreviations: DJGL, De Jong Gierveld Loneliness Scale; HADS-A, Hospital Anxiety and Depression Scale – Anxiety Subscale; MD, Mean Difference; MSPSS, Multidimensional Scale of Perceived Social Support; PHQ-9, Patient Health Questionnaire – 9 item; PSS, Perceived Stress Scale; PWB, Psychological Well-Being Scale; SCI, Sleep Condition Indicator; SWLS, Satisfaction with Life Scale; Tele-BA, Telephone-delivered behavioral activation; Tele-MF, Telephone-delivered mindfulness; Tele-BF, Telephone-delivered befriending; UCLA-LS, UCLA Loneliness Scale.

All p-values reported are non-adjusted.

[a]Linear mixed models (LMM) with maximum likelihood missing data treatment, controlling baseline outcome.

[b]Paired *t* test between scores at baseline and other assessment points were conducted for each intervention group separately. Results at 12 months are shown here.

[c]UCLA-LS score range 20-80, with 20 indicating low level of loneliness and 80 indicating high level of loneliness.

[d]DJGL score range 0-6, with 0 indicating no loneliness and 6 indicating severe loneliness.

[e]SCI score range 0-32, with 0 indicating poor sleep quality and 32 indicating good sleep quality.

[f]PWB score range 16-96, with 16 indicating low psychological well-being and 96 indicating high psychological well-being.

[g]MSPSS score range 12-84, with 12 indicating low perceived social support and 84 indicating high perceived social support.

[h]PHQ-9 score range 0-27, with 0 indicating no depressive symptoms and 27 indicating severe depressive symptoms.

[i]HADS-A score range 0-21, with 0 indicating no anxiety and 21 indicating severe anxiety.

[j]PSS score range 0-56, with 0 indicating no perceived stress and 56 indicating extremely high perceived stress.

[k]SWLS score range 5-35, with 5 indicating low life satisfaction and 35 indicating high life satisfaction.

© 2026 Tang VFY et al. *JAMA Network Open.*

eTable 5. Mixed-Effects Analysis at 12 months (Per-protocol)[a]

	Estimates across assessments, mean (95% CI) [b]						Tele-BA vs Tele-BF			Tele-MF vs Tele-BF			Overall between-group difference ***P* value**
Measures	Tele-BA (n=335)	*P* value	Tele-MF (n=460)	*P* value	Tele-BF (n=356)	*P* value	MD (95% CI)	Cohen d	MD *P* value	MD (95% CI)	Cohen d	MD *P* value	
**Primary Outcomes**													
UCLA-LS[c]	48.08 (47.35, 48.80)	<.001	48.23 (47.56, 48.89)	<.001	49.02 (48.39, 49.66)	<.001	-0.95 (-2.00, 0.11)	0.11	0.10	-0.79 (-1.80, 0.21)	0.11	0.18	<.001
DJGL[d]	3.89 (3.76, 4.01)	<.001	3.93 (3.82, 4.05)	<.001	3.96 (3.85, 4.07)	<.001	-0.07 (-0.25, 0.11)	0.06	0.98	-0.03 (-0.20, 0.14)	0.02	>.99	0.01
**Secondary Outcomes**													
SCI[e]	22.30 (21.61, 22.99)	0.08	21.78 (21.16, 22.40)	0.01	21.68 (21.08, 22.28)	0.60	0.62 (-0.38, 1.62)	0.12	0.41	0.10 (-0.85, 1.05)	0.09	>.99	0.004
PWB[f]	60.04 (59.28, 60.81)	<.001	59.63 (58.94, 60.33)	<.001	59.71 (59.04, 60.37)	<.001	0.34 (-0.77, 1.44)	0.19	>.99	-0.07 (-1.12, 0.98)	0.14	>.99	<.001
MSPSS[g]	43.47 (42.14, 44.80)	<.001	42.67 (41.45, 43.88)	<.001	42.42 (41.26, 43.58)	<.001	1.05 (-0.88, 2.98)	0.14	0.57	0.25 (-1.59, 2.08)	0.21	>.99	<.001
PHQ-9[h]	2.37 (2.07, 2.66)	0.69	2.51 (2.24, 2.77)	0.27	2.80 (2.55, 3.06)	0.01	-0.44 (-0.86, -0.01)	0.54	0.04	-0.30 (-0.70,0.11)	0.50	0.23	<.001
HADS-A[i]	1.96 (1.69, 2.23)	0.40	2.09 (1.85, 2.34)	0.13	2.27 (2.04, 2.51)	0.01	-0.31 (-0.71, 0.08)	0.50	0.17	-0.18 (-0.55, 0.19)	0.46	0.74	<.001
PSS[j]	13.93 (13.14, 14.72)	0.47	14.40 (13.67, 15.14)	0.60	13.86 (13.16, 14.55)	<.001	0.08 (-1.08, 1.23)	0.05	>.99	0.55 (-0.57, 1.67)	0.22	0.72	<.001
SWLS[k]	20.72 (20.33, 21.11)	<.001	20.55 (20.20, 20.91)	<.001	20.47 (20.13, 20.81)	<.001	0.25 (-0.31, 0.81)	0.14	0.86	0.09 (-0.46, 0.63)	0.17	>.99	0.002

Notes.

Abbreviations: DJGL, De Jong Gierveld Loneliness Scale; HADS-A, Hospital Anxiety and Depression Scale – Anxiety Subscale; MD, Mean Difference; MSPSS, Multidimensional Scale of Perceived Social Support; PHQ-9, Patient Health Questionnaire – 9 item; PSS, Perceived Stress Scale; PWB, Psychological Well-Being Scale; SCI, Sleep Condition Indicator; SWLS, Satisfaction with Life Scale; Tele-BA, Telephone-delivered behavioral activation; Tele-MF, Telephone-delivered mindfulness; Tele-BF, Telephone-delivered befriending; UCLA-LS, UCLA Loneliness Scale.

All p-values reported are non-adjusted.

[a]Linear mixed models (LMM) with maximum likelihood missing data treatment, controlling for age, gender, marital status, and baseline outcome;
Participants completed at least 75% of sessions and completed T1 follow-up assessments (n=631);
Participants completed at least 75% of sessions and completed T2 follow-up assessments (n=574);
Participants completed at least 75% of sessions and completed T3 follow-up assessments (n=505);
Participants completed at least 75% of sessions and completed T4 follow-up assessments (n=452);

[b]Paired *t* test between scores at baseline and other assessment points were conducted for each intervention group separately. Results at 12 months are shown here.

[c]UCLA-LS score range 20-80, with 20 indicating low level of loneliness and 80 indicating high level of loneliness.

[d]DJGL score range 0-6, with 0 indicating no loneliness and 6 indicating severe loneliness.

[e]SCI score range 0-32, with 0 indicating poor sleep quality and 32 indicating good sleep quality.

[f]PWB score range 16-96, with 16 indicating low psychological well-being and 96 indicating high psychological well-being.

[g]MSPSS score range 12-84, with 12 indicating low perceived social support and 84 indicating high perceived social support.

[h]PHQ-9 score range 0-27, with 0 indicating no depressive symptoms and 27 indicating severe depressive symptoms.

[i]HADS-A score range 0-21, with 0 indicating no anxiety and 21 indicating severe anxiety.

[j]PSS score range 0-56, with 0 indicating no perceived stress and 56 indicating extremely high perceived stress.

[k]SWLS score range 5-35, with 5 indicating low life satisfaction and 35 indicating high life satisfaction.

© 2026 Tang VFY et al. *JAMA Network Open.*
