# Supplementary material for: Behavioral Activation and Mindfulness Interventions in Reducing Loneliness and Improving Well-Being in Older Adults: The HEAL-HOA Randomized Clinical Trial
Source: JAMA Netw Open. 2026 Feb 4;9(2):e2557170. doi: 10.1001/jamanetworkopen.2025.57170 (PMC12873770; doi:10.1001/jamanetworkopen.2025.57170)
Supplement: Supplement 3. — Data Sharing Statement [file jamanetwopen-e2557170-s003.pdf]

Data Sharing Statement

Tang. Behavioral Activation and Mindfulness Interventions in Reducing Loneliness and Improving Well-Being in Older Adults: The HEAL-HOA Randomized Clinical Trial. *JAMA Netw Open*. Published February 04, 2026. doi:10.1001/jamanetworkopen.2025.57170

Data
Additional Information: The study was registered in the Clinical Trials Registry of the University of Hong Kong Clinical Trials Centre (number HKUCTR-2929, http://www.hkuctr.com/Study/Show/f3b3b973f3aa4bb68747cd39c011d802) and Chinese Clinical Trial Registry (ChiCTR2300072909, https://www.chictr.org.cn/showproj.html?proj=200473).
Data available: No

Additional Information
Explanation for why data not available: Data from this study will be made available upon reasonable request. For data access, please contact Chou KL and/or Tang VFY.
